# Time is of the essence when treating necrotizing soft tissue infections: a systematic review and meta-analysis

**DOI:** 10.1186/s13017-019-0286-6

**Published:** 2020-01-08

**Authors:** Femke Nawijn, Diederik P. J. Smeeing, Roderick M. Houwert, Luke P. H. Leenen, Falco Hietbrink

**Affiliations:** Department of Surgery, University Medical Center Utrecht, Heidelberglaan 100, 3584 CX Utrecht, The Netherlands

**Keywords:** Necrotizing soft tissue infection, Necrotizing fasciitis, Surgery, Debridement, Mortality, Amputation, Systematic review, Meta-analysis

## Abstract

**Background:**

Although the phrase “time is fascia” is well acknowledged in the case of necrotizing soft tissue infections (NSTIs), solid evidence is lacking. The aim of this study is to review the current literature concerning the timing of surgery in relation to mortality and amputation in patients with NSTIs.

**Methods:**

A systematic search in PubMed/MEDLINE, Embase, Cumulative Index to Nursing and Allied Health Literature (CINAHL), and the Cochrane Controlled Register of Trials (CENTRAL) was performed. The primary outcomes were mortality and amputation. These outcomes were related to the following time-related variables: (1) time from onset symptoms to presentation; (2) time from onset symptoms to surgery; (3) time from presentation to surgery; (4) duration of the initial surgical procedure. For the meta-analysis, the effects were estimated using random-effects meta-analysis models.

**Result:**

A total of 109 studies, with combined 6051 NSTI patients, were included. Of these 6051 NSTI patients, 1277 patients died (21.1%). A total of 33 studies, with combined 2123 NSTI patients, were included for quantitative analysis. Mortality was significantly lower for patients with surgery within 6 h after presentation compared to when treatment was delayed more than 6 h (OR 0.43; 95% CI 0.26–0.70; 10 studies included). Surgical treatment within 6 h resulted in a 19% mortality rate compared to 32% when surgical treatment was delayed over 6 h. Also, surgery within 12 h reduced the mortality compared to surgery after 12 h from presentation (OR 0.41; 95% CI 0.27–0.61; 16 studies included). Patient delay (time from onset of symptoms to presentation or surgery) did not significantly affect the mortality in this study. None of the time-related variables assessed significantly reduced the amputation rate. Three studies reported on the duration of the first surgery. They reported a mean operating time of 78, 81, and 102 min with associated mortality rates of 4, 11.4, and 60%, respectively.

**Conclusion:**

Average mortality rates reported remained constant (around 20%) over the past 20 years. Early surgical debridement lowers the mortality rate for NSTI with almost 50%. Thus, a sense of urgency is essential in the treatment of NSTI.

## Background

Necrotizing soft tissue infections (NSTIs) are notorious for their acute, aggressive, and rapidly progressive character. Of all NSTIs, necrotizing fasciitis is the most well known and most common NSTI; other NSTIs are myonecrosis and necrotizing cellulitis [1]. Mortality and amputation rates for NSTI are considered high, with described mortality rates varying between 6 and 33% [2–5]. Factors such as advanced age, female sex, multiple comorbidities, and sepsis upon presentation have previously been linked to increased mortality rates [2, 5, 6]. The bacteria causing NSTI can spread rapidly along the fascial planes; therefore, the saying “time is fascia” seems suitable. This resulted in the established belief that source control with early surgical resection of necrotic and infected tissue reduces progression of the infection and improves outcomes [1, 7]. However, the achievability of early treatment is sometimes hindered by a prolonged interval between the onset of symptoms and the patient seeking medical care (patient delay), or between hospital presentation and the eventual diagnosis (doctor delay) [8]. Furthermore, logistical challenges within hospitals might cause unwanted delays in treatment (system delay). In these cases, it is interesting to know if the prognosis can be predicted by the time frame in which the initial surgery is performed. If such a “golden” time frame exists, it could also indicate that when the delay was already too great, a higher mortality or amputation rate can be expected after initial surgery. There is still no consensus on a potential cut off point for such a time frame [9]. Multiple cohort studies have previously assessed the relation between surgical timing and mortality and amputation; however, a large number of studies are under-powered and were unable to reject the null hypothesis [10–14]. Therefore, the aim of this review was to analyze the current literature concerning the timing of surgery in relation to mortality and amputation in patients with necrotizing soft tissue infections.

## Review methods

A study protocol was developed a priori and submitted to PROSPERO for registration. This review is reported according to the Preferred Reporting Items for Systematic Reviews and Meta-Analyses (PRISMA) guidelines.

### Search and study selection

Published cohort studies and randomized controlled trials (RCT) reporting on mortality or amputation rates for NSTIs were included. These studies had to evaluate one of the following time-related variables: (1) time from onset symptoms to presentation; (2) time from onset symptoms to surgery; (3) time from presentation to surgery; and/or (4) duration of the initial surgical procedure. Studies written in English or Dutch were included. Conference abstracts, studies including pediatric patients, study protocols, reviews, animal studies, case reports, and studies reporting the results for the time variables for less than five patients were excluded.

Two reviewers (FN and DS) independently conducted a systematic search in PubMed/MEDLINE, Embase, Cumulative Index to Nursing and Allied Health Literature (CINAHL), and the Cochrane Controlled Register of Trials (CENTRAL) for articles published from inception of the databases up to October 29, 2019. The search syntax is available in Additional file 1. No filters were applied during the search. Titles and abstract were screened for potential eligible studies, after which duplicates were removed. The full texts of the potential eligible studies were screened by one reviewer (FN) for the reporting of one or more of the time-related variables. If the full-text article was not available online, attempts were made to request the article from the library or the authors. After screening the available full texts, the remaining articles were read in full to determine eligibility. In the case of uncertainty, the eligibility of a study was discussed between both reviewers. Disagreement of eligibility between reviewers was solved by discussion with a third independent reviewer (FH).

### Data extraction

The following data were extracted if available: first author, year of publication, country in which the study was conducted, study design, inclusion period, number of participating medical institutions, number of patients included, mean age of included patients, the anatomical regions affected by NSTI, inclusion and exclusion criteria, diagnostic criteria used for diagnosing NSTI (e.g., operative findings, histopathologic results, microbiology results, clinical signs during physical examination), time onset symptoms to presentation or surgery, time from presentation to surgery, duration of first surgery, mortality rate, and amputation rate. Data was extracted including the available odds ratio (OR), confidence intervals (CIs), and *p* values.

### Outcomes

The primary outcomes were mortality and amputation in NSTI patients. The previous mentioned time-related variables were assessed in relation to these outcomes. Due to heterogeneity in the reporting of the time variables, we assumed that time of presentation would be equal to time of hospital admittance or diagnosis, since NSTI patients often present septic and require immediate treatment hence the immediate hospital admission. We assumed that mortality rates reported in studies were in-hospital mortality rates, unless reported otherwise.

### Quality assessment

The methodological quality of the studies included in the meta-analysis was independently assessed by two reviewers (FN and DS). Since no suitable tool was available for this non-intervention-non-diagnostic study, a modified quality assessment tool based on the most applicable criteria from the Quality in Prognosis Studies (QUIPS) tool and Methodological Index for Non-Randomized Studies (MINORS) was used (Additional file 2) [15, 16]. Disagreement between reviewers during the quality assessment was resolved by discussion with a third independent reviewer (FH).

### Statistical analysis

Data management and statistical analysis were performed using Review Manager software (RevMan, version 5.3; Cochrane, Copenhagen, Denmark). Studies with data available for one or more of the time-related variables as categorical or dichotomous data in relation to either mortality or amputation were identified and included in the meta-analysis. If there was insufficient quantitative data to perform a meta-analysis for one or more of the time-related variables in relation to the outcomes, the time variable was assessed qualitatively. If required, data were manually categorized or calculated based on the available text or tables and was converted in the same units.

The stratification of time categories was data-driven. If the same time category (e.g., 6, 12, and 24 h) was compared in relation to mortality or amputation by ≥ 2 studies, this time categories were evaluated in a meta-analysis. Therefore, the available data per time category determined the stratification of the analyses for mortality and amputation. For the meta-analysis with amputation as outcome, the sample size was corrected to only include the patients with NSTI of the extremity or NSTI affecting multiple body areas. This was done to prevent underestimating the amputation rate if also NSTIs involving the trunk or perineum were included in the calculation of the amputation rate. The effect estimate for all analyses was an OR with a 95% CI calculated using the Mantel–Haenszel method. A *p* value < 0.05 in the overall effect *Z* test was considered statistically significant. Heterogeneity was evaluated using the following statistical measures: *τ*^2^, *I*^2^, and *χ*^2^. All analyses were performed using the random-effects model. Potential publication bias was assessed by eyeballing the funnel plots.

### Subgroup analyses

A priori, the following subgroup analyses were planned for each time-related variable if ≥ 2 studies were found for the subgroup analyses: (1) high-quality studies (a quality assessment score of 6 or higher out of a possible score of 8); (2) studies published in the last decade; (3) studies assessing NSTI of the entire body without excluding specific body regions; (4) studies that assessed all microbial NSTI entities without excluding specific microorganism.

## Review results

### Search

**Fig. 1:**
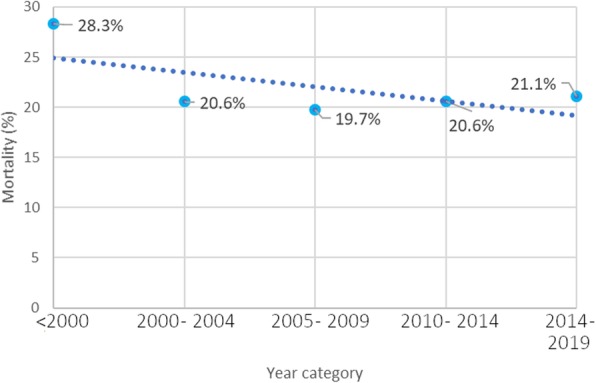
Historical cumulative mortality rates for necrotizing soft tissue infections based on included studies

**Fig. 2:**
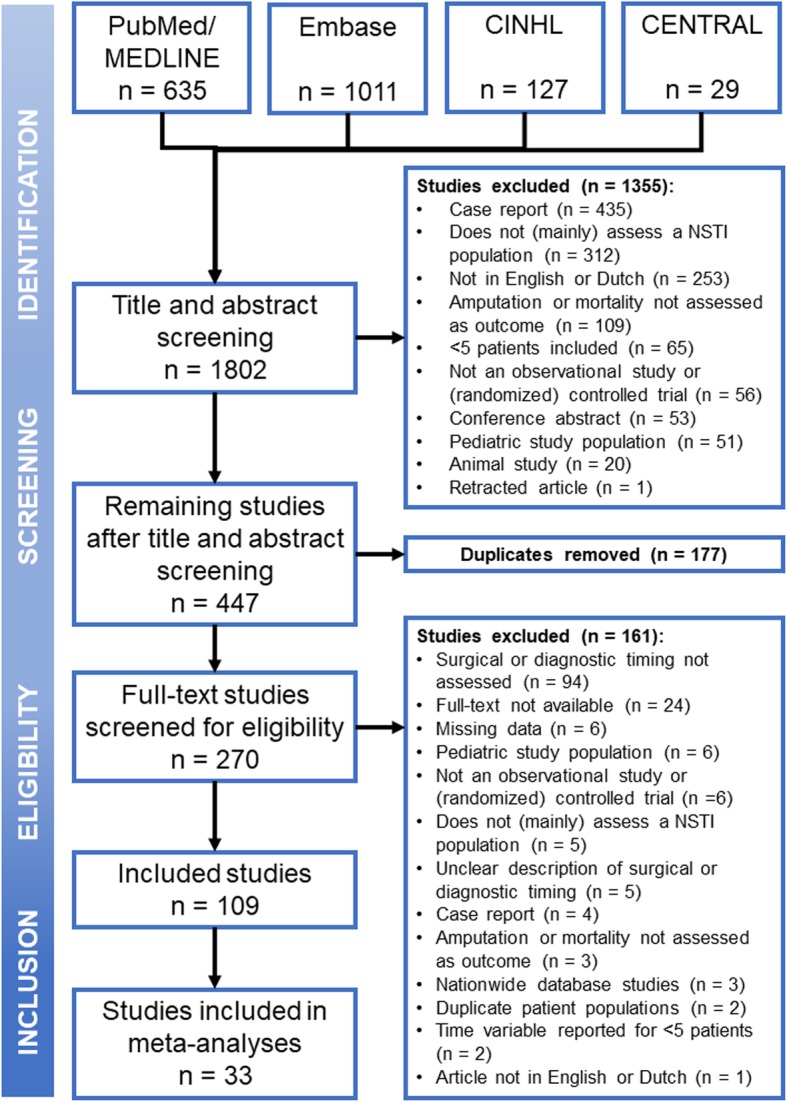
Flowchart of study inclusion process for meta-analysis of surgical timing of necrotizing soft tissue infections

After full text screening, 109 eligible studies were identified. The studies from Tsai et al. from 2004 [17] and 2009 [18] were excluded based on overlap in patient populations with a later published study from their group in 2010 [19]. The studies by Ahn et al., Holena et al., and Sugihara et al. were excluded, since they included patients from nationwide financial code-based databases without evident review of the included patient medical charts on content for eligibility [13, 20, 21]. All 109 articles combined; 6051 patients were included. Of these 6051 NSTI patients, 1277 patients died (21.1%) and 529 of the 2781 patients with NSTI of the extremity underwent an amputation (19.0%). Comparing mortality rates before and after 2000, there was a significant reduction in mortality from 28.3 to 20.6% (*p* = 0.004). However, average mortality rates reported remained constant (around 20%) over the past 20 years (Fig. 1). The baseline characteristics are summarized in Table 1. The elaborate baseline characteristics, study inclusion and exclusion criteria, the time-related variables assessed, and outcomes can be found in Additional file 3. A total of 33 studies were included for quantitative analysis. The selection process and reasons for exclusion can be found in Fig. 2.

**Table 1:** Baseline study characteristics of necrotizing soft tissue infection studies assessing surgical timing

	Eligible studies (*n* = 109)	Studies in meta-analyses (*n* = 33)
Publication year, *n* (%)		
1989 and older	8 (7)	3 (9)
1990–1999	7 (6)	4 (12)
2000–2009	29 (27)	7 (21)
2010–2019	65 (60)	19 (58)
Continent where study was performed^a^, *n* (%)		
Africa	8 (7)	0 (0)
Asia	42 (39)	14 (43)
Europe	23 (21)	7 (21)
North America	32 (30)	12 (36)
Oceania	3 (3)	0 (0)
South America	0 (0)	0 (0)
Type of study^b^, *n* (%)		
Retrospective cohort study	89 (90)	27 (93)
Prospective cohort study	9 (9)	2 (7)
Randomized controlled trial	1 (1)	0 (0)
Study period in years, median (IQR; range)	7 (5–11; 2–24)	6 (5–11; 2–16)
Number of participating medical institutions^a^, median (IQR; range)	1 (1–1; 1–6)	1 (1–1; 1–2)
Number of included patients per study, median (IQR; range)	35 (20–67; 5–472)	33 (20–84; 9–472)
Body regions affected by NSTI assed per study, *n* (%)		
Head and/or neck	9 (8)	1 (3)
Extremities	8 (8)	3 (9)
Trunk	2 (2)	1 (3)
Fournier	32 (29)	4 (12)
Full body	58 (53)	24 (73)

*IQR* interquartile range, *NSTI* necrotizing soft tissue infection

^a^1 missing case

^b^10 missing cases

### Baseline characteristics of studies in quantitative analysis

The 33 studies available for quantitative and thorough analysis included a combined number of 2123 NSTI patients with a mean age of 54 years. Of the 2123 patients, 417 patients (19.6%) died due to the NSTI. The number of patients included per study ranged between 9 and 472 patients. The majority of the studies included NSTI patients without having exclusion criteria for specific body regions affected (*n* = 23, 70%) (Table 1).

### Time from presentation at hospital to surgery

#### Surgery within 6 h

**Fig. 3:**
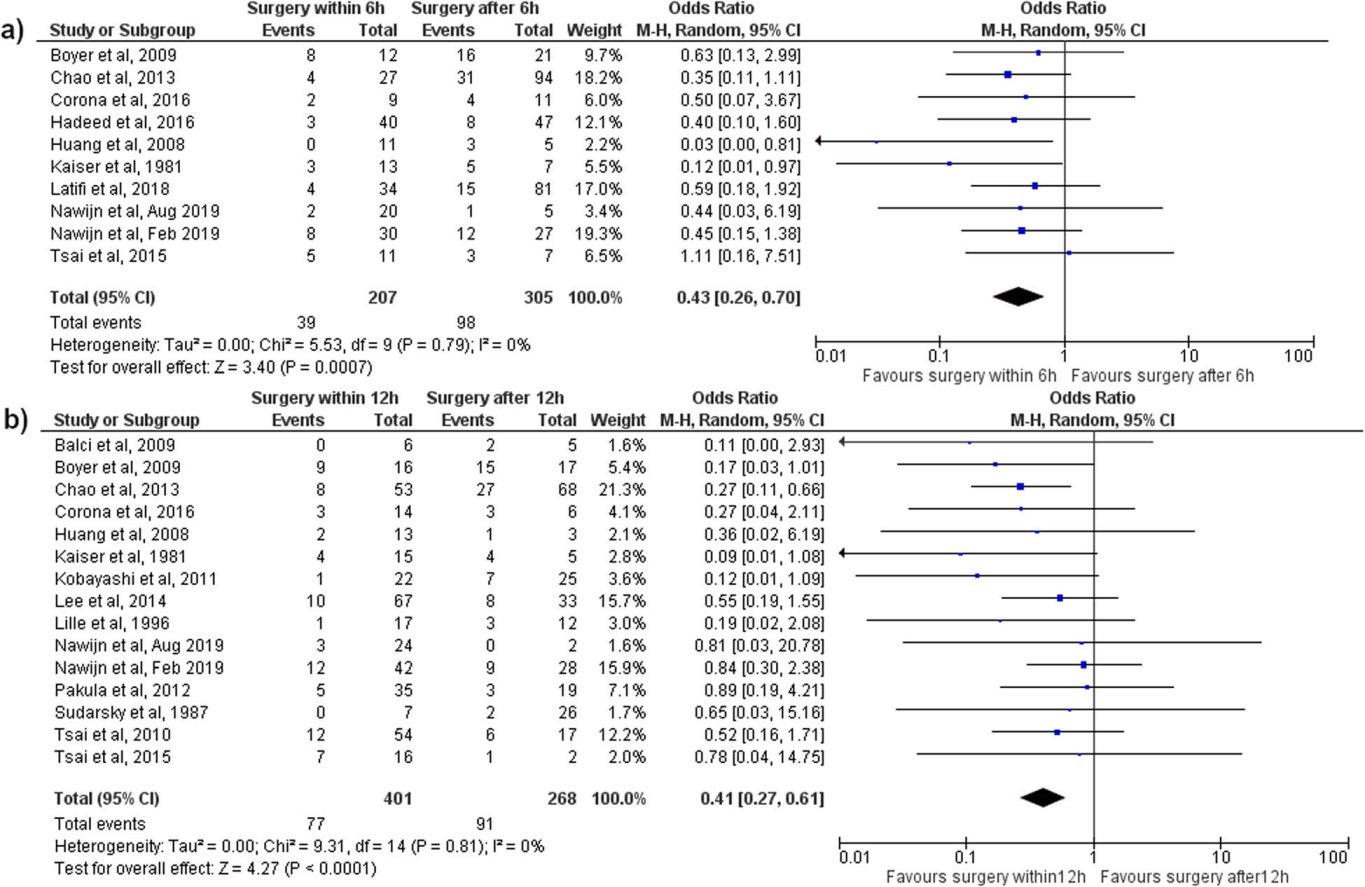
Mortality in a meta-analysis assessing time from presentation to surgery in necrotizing soft tissue patients. **a** Mortality in a meta-analysis comparing surgery within and after 6 h after presentation; **b** Mortality in a meta-analysis comparing surgery within and after 12 h after presentation

Ten (30%) of the 33 included studies reported the number of patients operated on within and after 6 h after presentation. The mortality was significantly lower for surgery within 6 h after presentation compared to surgical treatment delayed more than 6 h, with an OR of 0.43 (95% CI 0.26–0.70, *p* < 0.01) (Fig. 3a). Surgical treatment within 6 h resulted in a 19% mortality rate and surgical treatment after 6 h in a mortality rate of 32%. Surgery within 6 h did not result in a significant reduction in the amputation rate, with an OR of 0.68 (95% CI 0.34–1.39, *p* = 0.30) (Table 2 and Additional file 4).

**Table 2:** Results of meta-analyses assessing influence of surgical timing on outcomes in necrotizing soft tissue infections

Mortality analyses						
			Subgroup analyses			
Outcome	Events/total patients (*n*)	Result (OR, 95% CI)	High-quality studies^a^ (OR, 95% CI)	Studies published ≥ 2009^b^ (OR, 95% CI)	Studies without limitation on affected body region by NSTI^c^ (OR, 95% CI)	Studies without limitation based on specific microbial types of NSTI^d^ (OR, 95% CI)
Surgery within 6 h after presentation	137/512	**0.43 (0.26–0.70)**	**0.46 (0.27–0.80)**	**0.49 (0.30–0.82)**	**0.44 (0.25–0.75)**	**0.45 (0.25–0.79)**
Surgery within 12 h after presentation	168/669	**0.41 (0.27–0.61)**	**0.40 (0.23–0.69)**	**0.43 (0.28–0.67)**	**0.45 (0.29–0.70)**	**0.41 (0.22–0.74)**
Surgery within 24 h after presentation	271/1372	0.79 (0.52–1.20)	0.63 (0.29–1.34)	0.84 (0.52–1.37)	0.85 (0.53–1.38)	1.11 (0.77–1.60)
Surgery within 3 days after onset symptoms	33/172	0.40 (0.15–1.08)	0.41 (0.13–1.29)	0.35 (0.12–1.02)	0.46 (0.16–2.42)	0.13 (0.01–2.42)
Hospital admission within 3 days after onset symptoms	98/326	0.49 (0.16–1.44)	0.66 (0.15–2.83)	0.61 (0.17–2.24)	0.41 (0.08–2.13)	1.01 (0.37–2.74)
Amputation analyses						
			Subgroup analyses			
Outcome	Events/total patients (*n*)	Result (OR, 95% CI)	High-quality studies^a^ (OR, 95% CI)	Studies published ≥ 2009^b^ (OR, 95% CI)	Studies without limitation on affected body region by NSTI^c^ (OR, 95% CI)	Studies without limitation based on specific microbial types of NSTI^d^ (OR, 95% CI)
Surgery within 6 h after presentation	45/197	0.68 (0.34–1.39)	0.57 (0.23–1.42)	0.65 (0.31–1.38)	0.64 (0.30–1.38)	0.61 (0.28–1.32)
Surgery within 12 h after presentation	26/138	0.71 (0.28–1.82)	0.54 (0.11–2.54)	0.71 (0.25–1.98)	0.71 (0.24–2.11)	0.55 (0.19–1.54)
Surgery within 24 h after presentation	21/102	0.63 (0.20–2.05)	0.25 (0.04–1.60)	0.70 (0.19–2.58)	0.41 (0.08–2.26)	0.53 (0.14–2.06)

Results in bold are statistically significant results

*CI* confidence interval, *NSTI* necrotizing soft tissue infection, *OR* odds ratio

^a^Excluding Bair, Balci, Catena, Corona, Ferretti, George, Huang 2008, Kaiser, Kalaivani, Knutson, Lille, Liu, Mittapalli, Ogilvie, Pakula, Palmer, Park, Stephenson, Sudarsky, Tsai 2010, Tsai 2015, Wang, Yu

^b^Excluding Catena, Huang 2008, Kaiser, Knutson, Lille, Ogilvie, Palmer, Stephenson, Sudarsky, Wang, Yu

^c^Excluding Balci, Boyer, Corona, Ferretti, Huang 2008, Liu, Palmer, Stephenson, Sugihara, Wang

^d^Excluding Chao, Huang 2008, Knutson, Lee, Tsai 2010, Tsai 2015

#### Surgery within 12 h

Sixteen (48%) of the 33 included studies reported the number of patients operated on within and after 12 h after presentation. The mortality was significantly lower for surgery within 12 h after presentation compared to surgical treatment delayed more than 12 h, with an OR of 0.41 (95% CI 0.27–0.61, *p* < 0.01) (Fig. 3b). Surgical treatment within 12 h resulted in a 19% mortality rate and surgical treatment after 12 h in a mortality rate of 34%. Surgery within 12 h did not result in a significant lower amputation rate, with an OR of 0.71 (95% CI 0.28–1.82, *p* = 0.48) (Table 2 and Additional file 4).

#### Surgery within 24 h

Eighteen (55%) of the 33 included studies reported the number of patients operated on within and after 24 h after presentation. Analysis showed no significant reduction in the mortality or amputation rate between surgical treatment within or after 24 h, with an OR of 0.79 (95% CI 0.52–1.20, *p* = 0.26) for mortality and an OR of 0.63 (95% CI 0.20–2.05, *p* = 0.45) for amputation (Table 2 and Additional file 4).

### Time from onset symptoms to presentation at hospital

Forty-three studies included in the qualitative analysis reported on time from onset symptoms to presentation. The average time weighted by study sample sizes was 4.5 days (range 1.0–13.3 days). Since continuous independent variables cannot be used in meta-analyses, only studies with similar dichotomous variables were included in this meta-analysis. Eight (24%) of the 33 studies included for meta-analysis reported the number of patients presenting to the hospital within and after 3 days after the onset of the symptoms. Presentation to the hospital within 3 days after the onset of symptoms did not result in significant lower mortality than patients presenting after 3 days, with an OR of 0.49 (95% CI 0.16–1.44) (Table 2 and Additional file 4).

### Time from onset symptoms to surgery

Thirteen studies included in the qualitative analysis reported on time from onset symptoms to surgery. The average time weighted by study sample sizes was 4.6 days (range 2.1–7.5 days). Only studies with similar dichotomous variables were included in this meta-analysis. Three (9%) of the 33 included studies reported the number of patients operated on within and after 3 days after onset of symptoms. Surgery within 3 days after onset of symptoms did not result in significant lower mortality than patients operated after 3 days, with an OR of 0.40 (95% CI 0.15–1.08) (Table 2 and Additional file 4).

### Duration of first surgery

Only three studies reported on the duration of the first surgery. Corman et al. found a mortality rate of 4% (1 out of 26 patients) with an associated mean duration of the initial surgery of 78 min; Elskaket et al. reported a mortality rate of 11.4% (5 out of 44 patients) associated with a mean duration of the initial surgery of 81 min, while Hong et al. reported a mortality rate of 60% (9 out of 15 patients) associated with a mean duration of the initial surgery of 102 min.

### Quality assessment

The elaborate results of the quality assessment for each study can be found in Additional file 5. The mean quality score was 5 ± 2. Ten (30%) studies scored 6 or higher, indicating high quality.

### Subgroup analyses

The subgroup analyses either using only studies published in the last decade, studies assessing NSTI of the entire body without excluding specific body regions, or studies that assessed all microbial NSTI entities without only including a specific microorganism did not result in new results. No outcomes changed direction or significance (Table 2 and Additional file 4).

### Assessment of publication bias

**Fig. 4:**
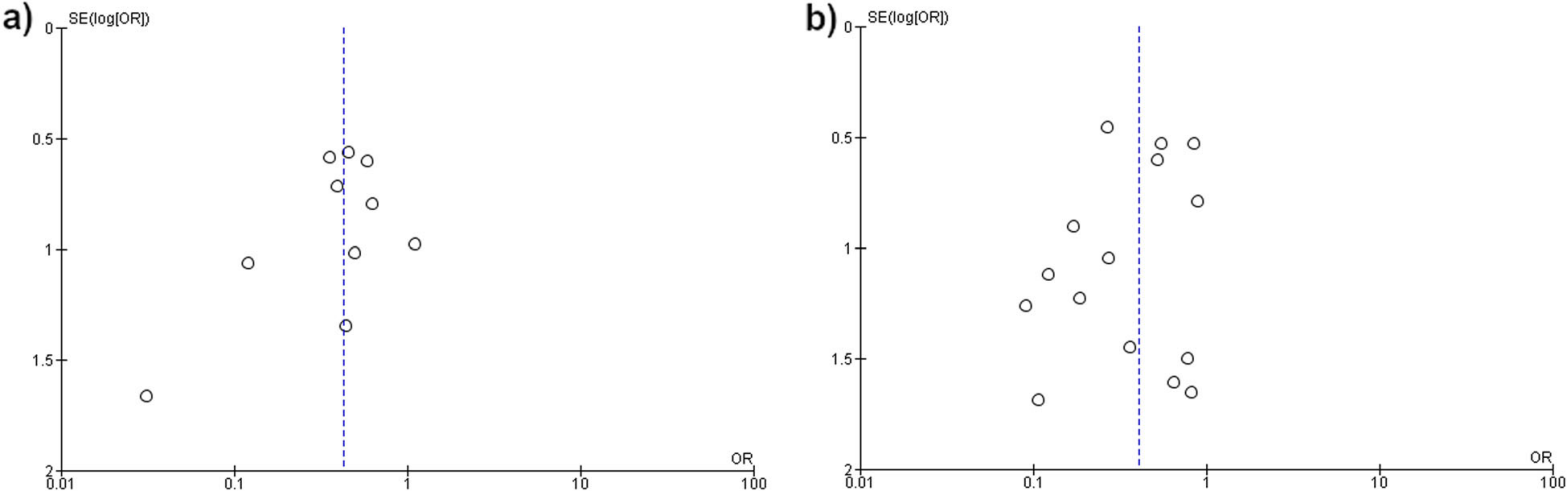
Funnel plot of meta-analysis assessing surgical timing and mortality in necrotizing soft tissue infections. **a** Funnel plot for meta-analysis comparing mortality in necrotizing soft tissue infection patients operated within or after 6 h after presentation; **b** Funnel plot for meta-analysis comparing mortality in necrotizing soft tissue infection patients operated within or after 12 h after presentation

The funnel plot for the analysis of time from presentation to surgery within and after 6 and 12 h in relation to mortality are presented in Fig. 4. Upon eyeballing the funnel plots, both showed relative symmetry indicating a low risk of publication bias in these meta-analyses.

## Discussion

This study clearly shows that the average mortality rate for NSTI did not improve over the past 20 years. Timely initial surgery after presentation to the hospital for NSTI cuts mortality almost in half. This stresses the need for early surgical treatment of all NSTIs.

There is only one similar meta-analysis published that assesses the time to surgery for NSTIs. Gelbard et al. pooled the results from six studies and found an OR of 0.43 (95% CI 0.24–0.78) in favor of surgery within 12 h (13% mortality) compared to surgery after 12 h from presentation (26% mortality) [8]. Our study shows a similar reduction in mortality if the initial surgery is performed within 12 h after presentation (19 vs. 34%), but even more, also found such an association for surgery within 6 h (19 vs. 32%). Based on our results, initial surgery within 12 h should be regarded as the minimal “golden” time frame for operating patients with NSTIs, while surgery within 6 h might be strongly preferred. However, based on these analyses, it is difficult to give a prognosis for the patients operated on between 6 and 12 h. Based on the analyses comparing surgery within and after 12 h, these patients were less likely to die (OR 0.41 for surgery within 12 h; 95% CI 0.27–0.61), while in the analysis comparing surgery within and after 6 h, this group of patients had worse outcomes (OR 0.43 for surgery within 6 h; 95% CI 0.26–0.70). Although surgery within 12 h is essential, surgery within 6 h might be beneficial. However, to determine a more exact cutoff point for the “golden” time frame, more research is necessary.

Patient delay (time from onset of symptoms to surgery) did not seem to affect mortality, although availability and robustness of the data for this part of the question was limited. Nevertheless, based on the presented mortality data in this review, the time from presentation to surgery (which encompasses both doctor delay and part of the system delay) has significant effect on outcome. On the other hand, this study did not find an association between timing of surgery and the amputation rate, indicating that other factors, such as comorbidities, the local situation of the tissue (e.g., the presence of bullae), or the severity of disease (e.g., severe sepsis), are more predictive for amputation [22, 23]. However, those factors were outside the scope of this review.

The goal of the initial surgical procedure for NSTIs is to gain control and prevent further (trans-fascial and hematogenous) spreading of the infection by complete debridement of all the infected and necrotic tissue [1, 9]. Sarani et al. suggested that each hour delay of surgical treatment can lead to a local spread of the infection as fast as an inch per hour and results in higher chances of systematic spread [24]. Early surgical treatment does not only reduce the mortality rate, but several studies also found that it can reduce the risk of septic shock, number of surgical debridement, and the length of hospital stay [14, 25]. The exact pathophysiology behind the rapid spread of bacteria across the fascia is still poorly understood. However, it is thought that especially during NSTIs the microbial virulence caused by the toxins produced by the involved bacteria outweighs the host defense system providing the opportunity for rapid spreading of the infection [24, 26]. Early resection of necrotic and infected tissue results in a lower microbial load. As a result, the immune system combined with broad spectrum antibiotics has better odds at controlling the infection [1, 27]. Thus, time is of the essence.

However, clinical implementation of the desired urgent debridement is often hindered by multiple factors. First, patient delay is a problem not easily influenced by medical personnel. The time a patient waits before seeking medical care is dependent on a wide variety of clinical, economic, and social factors. The physical and financial access to emergency care, the nature of the acute illness, the underlying chronic comorbidities, and understanding of the severity of symptoms all influence the likelihood of a patient seeking emergency care [28].

Next, doctor delay is a well-known problem for this disease. Before NSTI can be treated, the accurate diagnosis must be made. Awareness of NSTI is frequently described as low, due to its low incidence, compared to non-necrotizing soft tissue infections with a higher a priori chance such as cellulitis and erysipelas [3, 29]. Furthermore, symptoms of NSTI mimic those of cellulitis and erysipelas and no pathognomic symptoms for NSTI are known [23, 30, 31]. Wong et al. developed the laboratory risk indicator for necrotizing fasciitis (LRINEC) score to help physicians with identifying NSTIs [32]. However, a meta-analysis performed by Fernando et al. showed that this is a suboptimal score for identifying patients with NSTI due to its low sensitivity [30]. The substantial problem of misdiagnosing is illustrated in a systematic review by Goh et al. They reported that 71.4% of the NSTIs were initially misdiagnosed and that the mortality rate increased with the percentage of initially missed diagnoses [23]. Interoperative diagnostic accuracy can be increased by using the method of triple diagnostics. In the case of ambivalent signs of NSTI upon intra-operative macroscopic evaluation, samples should be taken for intra-operative assessment of fresh frozen sections and Gram stains. Based on those results, the NSTI diagnosis can be confirmed or waived [7, 33]. A solution for improving pre-operative diagnostics is a strongly recommended focus for future studies.

Finally, the medical system should be organized with enough surgical capacity to prevent system delay. After the accurate diagnosis is made, the logistics needs to be in place to facilitate urgent surgical debridement. The initial debridement for NSTI holds the highest surgical priority. McIsaac et al. reported that 27% of the urgent or emergency surgeries at their hospital with the highest priority were delayed beyond the waiting time appointed to surgeries with the highest priority. The main reasons for the delays were unavailability of surgeons, followed by unavailability of resources such as operating rooms [34]. Improving the availability of the appropriate surgeons and resources at the presenting hospital is crucial, since transfer, even to a center specialized in NSTIs, increases the delay and therefore the risk at mortality [21]. To improve immediate availability of the appropriate resources, the system using 24/7 in-house attending surgeon and the 24/7 readiness of an operating room could significantly decrease the time to surgery and mortality [35, 36].

Not only the time to surgery influences the outcomes, but shorter operative times of emergency surgeries are also associated with less postoperative complications [37]. Matsuyama et al. reported that the mortality and morbidity are significantly lower if emergency surgeries in adults were completed within 120 min, and Kaushal-Deep et al. reports better outcomes if operative times are less than 100 min for pediatric emergency surgeries [37, 38]. In severely physiologically compromised trauma patients, the damage control strategy is indicated if the operative time would be longer than 90 min [39]. Unfortunately, our study is unable to comment on the ideal duration of the initial debridement for NSTI and remains therefore unknown. However, since most NSTI patients are severely physiologically compromised, short and efficient debridements might be recommended, as a major difference in mortality rate was noted between the published results of patients with an operating time shorter and longer than 90 min. The risk at more postoperative complications associated with longer operative times should be considered when skin-sparing debridement for NSTIs is contemplated [37, 40]. Therefore, the clinical condition of the patient should determine the course of actions and surgical strategy.

The limitations of this study need to be kept in mind during the interpretation of the results. For example, we were unable to vary between time from diagnosis to surgery and time from presentation to surgery. The time from presentation to diagnosis is often underreported and could not be assessed. Furthermore, even though we used a broad search, there is still a possibility of missing studies. Finally, for the interpretation of the cumulative mortality rates, it should be kept in mind that the included studies used different and sometimes very specific inclusion and exclusion criteria, limiting the generalizability of mortality rates to the entire NSTI population. For example, eight studies excluded patients that did not undergo surgery, which indicates that those patients were unsuitable for surgery (i.e., based on severity of illness or patients’ wishes) [10, 41–47]. Excluding these patients from the mortality rate could result in a seemingly better mortality rate than the reality, since these patients are likely to have died of NSTI. The strength of this meta-analysis is the relatively low heterogeneity in the meta-analysis, and the risk at publication bias is estimated to be limited. Furthermore, this meta-analysis contributes to solving the problem of underpowered studies, which is especially relevant in the field of NSTI research. The incidence of NSTI has been estimated to be 3.64 per 100,000 person years; this suggests that most single-center NSTI study would automatically be underpowered due to the limited incidence of NSTI to that hospital [3]. Therefore, meta-analyses remain an efficient way of increasing the body of evidence if only studies with limited sample sizes are available.

## Conclusion

Average mortality rates reported remained constant (around 20%) over the past 20 years. Surgical debridement as soon as possible lowers the mortality rate for NSTI with almost 50%. However, early surgical treatment did not reduce the amputation rate. Nevertheless, this systematic review and meta-analysis show that early surgical treatment of NSTIs within 12 h is essential for reducing the mortality rate, while surgical treatment within 6 h might even further improve outcomes.

## Supplementary information

**Additional file 1:** Search syntax for systematic review assessing surgical timing in relation to mortality and amputation due to necrotizing soft tissue infections.

**Additional file 2:** Quality assessment tool for systematic review assessing surgical timing in relation to mortality and amputation due to necrotizing soft tissue infections.

**Additional file 3:** Extracted data from eligible studies assessing surgical timing related to mortality and amputation due to necrotizing soft tissue infection.

**Additional file 4:** Forest plots for all (subgroup) analyses assessing surgical timing in relation to mortality and amputation due to necrotizing soft tissue infections.

**Additional file 5:** Results from quality assessment of articles included in meta-analyses assessing surgical timing in relation to mortality and amputation due to necrotizing soft tissue infections.

## Data Availability

The data supporting the conclusions of this article are included within the article and its additional files.

## Abbreviations

CENTRAL: Cochrane Controlled Register of Trails
CI: Confidence interval
CINAHL: Cumulative Index to Nursing and Allied Health Literature
IQR: Interquartile range
MD: Mean difference
MINORS: Methodological Index for Non-Randomized Studies
NSTI: Necrotizing soft tissue infection
OR: Odds ratio
PRISMA: Preferred Reporting Items for Systematic Reviews and Meta-analyses
QUIPS: Quality in Prognosis Studies
RCT: Randomized controlled trials
SD: Standard deviation

## References

[1] Sartelli M, Guirao X, Hardcastle TC, Kluger Y, Boermeester MA, Raşa K (2018). 2018 WSES/SIS-E consensus conference: recommendations for the management of skin and soft-tissue infections. World J Emerg Surg..

[2] Krieg A, Dizdar L, Verde PE, Knoefel WT (2014). Predictors of mortality for necrotizing soft-tissue infections: a retrospective analysis of 64 cases. Langenbeck’s Arch Surg..

[3] Audureau E, Hua C, de Prost N, Hemery F, Decousser JW, Bosc R (2017). Mortality of necrotizing fasciitis: relative influence of individual and hospital-level factors, a nationwide multilevel study, France, 2007–12. Br J Dermatol..

[4] Kao LS, Lew DF, Arab SN, Todd SR, Awad SS, Carrick MM (2011). Local variations in the epidemiology, microbiology, and outcome of necrotizing soft-tissue infections: a multicenter study. Am J Surg..

[5] Nawijn F, Wassenaar ECE, Smeeing DPJ, Vlaminckx BJM, Reinders JSK, Wille J (2019). Exhaustion of the immune system by group A Streptococcus necrotizing fasciitis: the occurrence of late secondary infections in a retrospective study. Trauma Surg Acute Care Open..

[6] Anaya D, McMahon K, Nathens A, Sullivan S, Foy H, Bulger E (2005). Predictors of mortality and limb loss in necrotizing soft tissue infections. Arch Surg..

[7] Nawijn F, Houwert RM, van Wessem KPJ, Simmermacher RKJ, Govaert GAM, van Dijk MR (2019). A 5-year evaluation of the implementation of triple diagnostics for early detection of severe necrotizing soft tissue disease: a single-center cohort study. World J Surg..

[8] Gelbard RB, Ferrada P, Yeh DD, Williams BH, Loor M, Yon J (2018). Optimal timing of initial debridement for necrotizing soft tissue infection: a practice management guideline from the Eastern Association for the Surgery of Trauma. J Trauma Acute Care Surg..

[9] Stevens DL, Bryant AE (2017). Necrotizing soft-tissue infections. N Engl J Med..

[10] Chao WN, Tsai CF, Chang HR, Chan KS, Su CH, Lee YT (2013). Impact of timing of surgery on outcome of Vibrio vulnificus-related necrotizing fasciitis. Am J Surg..

[11] Bair MJ, Chi H, Wang WS, Hsiao YC, Chiang RA, Chang KY (2009). Necrotizing fasciitis in southeast Taiwan: clinical features, microbiology, and prognosis. Int J Infect Dis..

[12] Huang KF, Hung MH, Lin YS, Lu CL, Liu C, Chen CC (2011). Independent predictors of mortality for necrotizing fasciitis: a retrospective analysis in a single institution. J Trauma Inj Infect Crit Care..

[13] Sugihara T, Yasunaga H, Horiguchi H, Fujimura T, Ohe K, Matsuda S (2012). Impact of surgical intervention timing on the case fatality rate for Fournier’s gangrene: an analysis of 379 cases. BJU Int.

[14] Latifi R, Patel AS, Samson DJ, Tilley EH, Gashi S, Bergamaschi R (2019). The roles of early surgery and comorbid conditions on outcomes of severe necrotizing soft-tissue infections. Eur J Trauma Emerg Surg..

[15] Hayden J, van der Windt D, Cartwright J, Côté P, Bombardier C (2013). Assessing bias in studies of prognostic factors. Ann Intern Med..

[16] Slim K, Nini E, Forestier D, Kwiatkowski F, Panis Y, Chipponi J (2003). Methodological Index for Non-Randomized Studies (Minors): development and validation of a new instrument. Anz J Surg..

[17] Tsai Y, Huang T, Hsu R, Weng Y, Hsu W, Huang K (2004). Systemic vibrio infection presenting as necrotizing fasciitis and sepsis: a series of thirteen cases. J Bone Jt Surg - Am Vol..

[18] Tsai Y-H, Huang T-J, Hsu RW-W, Weng Y-J, Hsu W-H, Huang K-C (2009). Necrotizing soft-tissue infections and primary sepsis caused by Vibrio vulnificus and Vibrio cholerae non-O1. J Trauma..

[19] Tsai Y-H, Hsu RW-W, Huang K-C, Huang T-J (2010). Laboratory indicators for early detection and surgical treatment of vibrio necrotizing fasciitis. Clin Orthop Relat Res..

[20] Ahn J, Raspovic K, Liu G, Lavery L, La Fontaine J, Nakonezny P, et al. Lower extremity necrotizing fasciitis in diabetic and nondiabetic patients: mortality and amputation. Int J Low Extrem Wounds. 2019:1–8. 10.1177/1534734619836464 30929530

[21] Holena DN, Mills AM, Carr BG (2011). Transfer status: a risk factor for mortality in patients with necrotizing fasciitis. Surgery..

[22] Khamnuan P, Chongruksut W, Jearwattanakanok K, Patumanond J, Tantraworasin A (2015). Necrotizing fasciitis: epidemiology and clinical predictors for amputation. Int J Gen Med..

[23] Goh T, Goh L, Ang C, Wong C (2014). Early diagnosis of necrotizing fasciitis. Br J Surg..

[24] Sarani B, Strong M, Pascual J, Schwab CW (2009). Necrotizing fasciitis: current concepts and review of the literature. J Am Coll Surg..

[25] Kobayashi L, Konstantinidis A, Shackelford S, Chan L, Talving P, Inaba K (2011). Necrotizing soft tissue infections: delayed surgical treatment is associated with increased number of surgical debridements and morbidity. J Trauma - Inj Infect Crit Care..

[26] Young MH, Aronoff DM, Engleberg NC (2005). Necrotizing fasciitis: pathogenesis and treatment. Expert Rev Anti Infect Ther..

[27] Bonne SL, Kadri SS (2017). Evaluation and management of necrotizing soft tissue infections. Infect Dis Clin North Am..

[28] Rucker DW, Brennan TA, Burstin HR (2001). Delay in seeking emergency care. Acad Emerg Med..

[29] Miller LG, Eisenberg DF, Liu H, Chang CL, Wang Y, Luthra R (2015). Incidence of skin and soft tissue infections in ambulatory and inpatient settings, 2005-2010. BMC Infect Dis..

[30] Fernando S, Tran A, Cheng W, Rochwerg B, Kyeremanteng K, Seely A (2019). Necrotizing soft tissue infections: diagnostic accuracy of physical examination, imaging, and LRINEC score. Ann Surg..

[31] Wong C-H, Chang H-C, Pasupathy S, Khin L-W, Tan J-L, Low C-O (2003). Necrotizing fasciitis: clinical presentation, microbiology, and determinants of mortality. J Bone Joint Surg Am..

[32] Wong CH, Khin LW, Heng KS, Tan KC, Low CO (2004). The LRINEC (Laboratory Risk Indicator for Necrotizing Fasciitis) score: a tool for distinguishing necrotizing fasciitis from other soft tissue infections. Crit Care Med..

[33] Hietbrink F, Bode LG, Riddez L, Leenen LPH, van Dijk MR (2016). Triple diagnostics for early detection of ambivalent necrotizing fasciitis. World J Emerg Surg..

[34] McIsaac DI, Abdulla K, Yang H, Sundaresan S, Doering P, Vaswani SG (2017). Association of delay of urgent or emergency surgery with mortality and use of health care resources: a propensity score-matched observational cohort study. Cmaj..

[35] van der Vliet QMJ, van Maarseveen OEC, Smeeing DPJ, Houwert RM, van Wessem KJP, Simmermacher RKJ (2019). Severely injured patients benefit from in-house attending trauma surgeons. Injury..

[36] Daniel VT, Rushing AP, Ingraham AM, Ricci KB, Paredes AZ, DIaz A (2019). Association between operating room access and mortality for life-threatening general surgery emergencies. J Trauma Acute Care Surg..

[37] Matsuyama T, Iranami H, Fujii K, Inoue M, Nakagawa R, Kawashima K (2013). Risk factors for postoperative mortality and morbidities in emergency surgeries. J Anesth..

[38] Kaushal-Deep S, Lodhi A, Chana R (2019). Journal of Postgraduate Medicine Wolters Kluwer–Medknow Publications. A prospective study of evaluation of operative duration as a predictor of mortality in pediatric emergency surgery: concept of 100 minutes laparotomy in resource-limited setting. J Postgr Med..

[39] Lamb CM, Macgoey P, Navarro AP, Brooks AJ (2014). Damage control surgery in the era of damage control resuscitation. Br J Anaesth..

[40] Tom LK, Wright TJ, Horn DL, Bulger EM, Pham TN, Keys KA (2016). A skin-sparing approach to the treatment of necrotizing soft-tissue infections: thinking reconstruction at initial debridement. J Am Coll Surg..

[41] Park SJ, Kim DH, Choi CI, Yun SP, Kim JH, Seo H (2016). Il, et al. Necrotizing soft tissue infection: analysis of the factors related to mortality in 30 cases of a single institution for 5 years. Ann Surg Treat Res..

[42] Eggerstedt M, Gamelli RL, Mosier MJ (2015). The care of necrotizing soft-tissue infections: patterns of definitive intervention at a large referral center. J Burn Care Res..

[43] Okoye O, Talving P, Lam L, Smith J, Teixeira PG, Inaba K (2013). Timing of redebridement after initial source control impacts survival in necrotizing soft tissue infection. Am Surg..

[44] Yu K-H, Ho H-H, Chen J-Y, Luo S-F (2004). Gout complicated with necrotizing fasciitis—report of 15 cases. Rheumatology (Oxford)..

[45] Czymek R, Kujath P, Bruch H-P, Pfeiffer D, Nebrig M, Seehofer D (2013). Treatment, outcome and quality of life after Fournier’s gangrene: a multicentre study. Colorectal Dis..

[46] Hadeed GJ, Smith J, O’Keeffe T, Kulvatunyou N, Wynne JL, Joseph B (2016). Early surgical intervention and its impact on patients presenting with necrotizing soft tissue infections: a single academic center experience. J Emerg Trauma Shock..

[47] George ME, Rueth NM, Skarda DE, Chipman JG, Quickel RR, Beilman GJ (2009). Hyperbaric oxygen does not improve outcome in patients with necrotizing soft tissue infection. Surg Infect (Larchmt)..

