# Supplementary material for: Time is of the essence when treating necrotizing soft tissue infections: a systematic review and meta-analysis
Source: World J Emerg Surg. 2020 Jan 8;15:4. doi: 10.1186/s13017-019-0286-6 (PMC6950871; doi:10.1186/s13017-019-0286-6)
Supplement: Supplementary file 1 — Additional file 1: Search syntax for systematic review assessing surgical timing in relation to mortality and amputation due to necrotizing soft tissue infections. [file 13017_2019_286_MOESM1_ESM.pdf]

Additional file 1 Search syntax for systematic review assessing surgical timing in relation to mortality and amputation due to necrotizing soft tissue infections

PubMed/MEDLINE syntax (n = 635)

(fasciitis, necrotizing [MESH Terms] OR necrotizing fasciitis[Title/Abstract] OR necrotizing soft tissue infection[Title/Abstract] OR gas gangrene[Title/Abstract] OR Fournier’s gangrene[Title/Abstract] OR severe necrotizing soft tissue disease[Title/Abstract])

AND

((Time-to-treatment [MESH Term] OR Operative Time [MESH Term] OR time[Title/Abstract] OR hours[Title/Abstract] OR days[Title/Abstract]) AND (Surgical Procedures, operative[MESH Terms] OR Surgery [MESH Terms] OR Debridement [MESH Terms] OR surgery[Title/Abstract] OR debridement[Title/Abstract] OR operation[Title/Abstract]))

NOT

case report[Title/Abstract]

Embase syntax (n = 1011)

(‘necrotizing fasciitis’/exp OR ‘necrotizing fasciitis’:ti,ab OR ‘necrotizing soft tissue infection’:ti,ab OR ‘gas gangrene’:ti,ab OR ‘Fournier gangrene’:ti,ab OR ‘severe necrotizing soft tissue disease’:ti,ab)

AND

((‘time to treatment’/exp OR ‘operation duration’/exp OR ‘time’:ti,ab OR ‘hours’:ti,ab OR ‘days’:ti,ab) AND (‘debridement’/exp OR ‘surgery’/exp OR ‘surgery’:ti,ab OR ‘debridement’:ti,ab OR ‘operation’:ti,ab))

NOT

‘case report’:ti,ab

CINAHL syntax (n=127)

(MH fasciitis, necrotizing OR TI necrotizing fasciitis OR AB necrotizing fasciitis OR TI necrotizing soft tissue infection OR AB necrotizing soft tissue infection OR TI gas gangrene OR AB gas gangrene OR TI Fournier’s gangrene OR AB Fournier’s gangrene OR TI severe necrotizing soft tissue disease OR AB severe necrotizing soft tissue disease)

AND

(MH Time-to-treatment OR MH Operative Time OR TI time OR AB time OR TI hours OR AB hours OR TI days OR AB days) AND (MH Surgical Procedures, operative OR MH Surgery OR MH Debridement OR TI surgery OR AB surgery OR TI debridement OR AB debridement OR TI operation OR AB operation)

NOT

(TI case report OR AB case report)

CENTRAL syntax (n=29)

(MH ‘necrotizing fasciitis’ OR necrotizing fasciitis:ti,ab OR necrotizing soft tissue infection:ti,ab OR gas gangrene:ti,ab OR Fournier gangrene:ti,ab OR severe necrotizing soft tissue disease:ti,ab)

AND

((MH ‘time to treatment’ OR MH ‘operation duration’ OR time:ti,ab OR hours:ti,ab OR days:ti,ab) AND (MH

‘debridement’ OR MH ‘surgery’ OR surgery:ti,ab OR debridement:ti,ab OR operation:ti,ab))
