# Supplementary material for: Time is of the essence when treating necrotizing soft tissue infections: a systematic review and meta-analysis
Source: World J Emerg Surg. 2020 Jan 8;15:4. doi: 10.1186/s13017-019-0286-6 (PMC6950871; doi:10.1186/s13017-019-0286-6)
Supplement: Supplementary file 2 — Additional file 2: Quality assessment tool for systematic review assessing surgical timing in relation to mortality and amputation due to necrotizing soft tissue infections. [file 13017_2019_286_MOESM2_ESM.pdf]

Additional file 2 Quality assessment tool for systematic review assessing surgical timing in relation to mortality and amputation due to necrotizing soft tissue infections

Points	Prospective collection of data	Inclusion and exclusion criteria	Diagnostic criteria for necrotizing soft tissue infections used	Definition of the outcome
**2**	Prospective	Inclusion and exclusion criteria are adequately described	Macroscopic findings fascia, histopathology results and/or microbiology results	A clear definition of outcome is provided, including duration of follow-up period
**1**	Retrospective	Unclear or poor described inclusion and/or exclusion criteria	Only clinical signs	A definition of outcome is provided without reporting duration of follow-up period (including “in-hospital mortality” without reporting length of hospital stay)
**0**	Not reported	Not reported	Not reported	Not reported
