# Supplementary material for: Time is of the essence when treating necrotizing soft tissue infections: a systematic review and meta-analysis
Source: World J Emerg Surg. 2020 Jan 8;15:4. doi: 10.1186/s13017-019-0286-6 (PMC6950871; doi:10.1186/s13017-019-0286-6)

Additional file 4 Forrest plots for all (subgroup) analyses assessing surgical timing in relation to mortality and amputation due to necrotizing soft tissue infections

Surgery within 6 hours after presentation - mortality as outcome

	Surgery within 6h		Surgery after 6h			Odds Ratio
Study or Subgroup	Events	Total	Events	Total	Weight	M-H, Random, 95% CI
Boyer et al, 2009	8	12	16	21	9.7%	0.63 [0.13, 2.99]
Chao et al, 2013	4	27	31	94	18.2%	0.35 [0.11, 1.11]
Corona et al, 2016	2	9	4	11	6.0%	0.50 [0.07, 3.67]
Hadeed et al, 2016	3	40	8	47	12.1%	0.40 [0.10, 1.60]
Huang et al, 2008	0	11	3	5	2.2%	0.03 [0.00, 0.81]
Kaiser et al, 1981	3	13	5	7	5.5%	0.12 [0.01, 0.97]
Latifi et al, 2018	4	34	15	81	17.0%	0.59 [0.18, 1.92]
Nawijn et al, Aug 2019	2	20	1	5	3.4%	0.44 [0.03, 6.19]
Nawijn et al, Feb 2019	8	30	12	27	19.3%	0.45 [0.15, 1.38]
Tsai et al, 2015	5	11	3	7	6.5%	1.11 [0.16, 7.51]
**Total (95% CI)**		**207**		**305**	**100.0%**	**0.43 [0.26, 0.70]**
Total events	39		98			

Heterogeneity: Tau² = 0.00; Chi² = 5.53, df = 9 (P = 0.79); I² = 0%
Test for overall effect: Z = 3.40 (P = 0.0007)

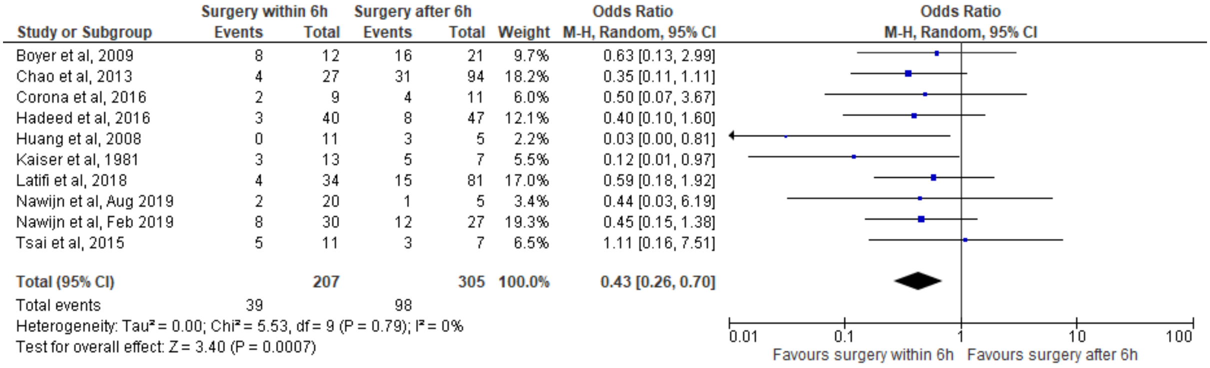

Subgroup analysis: High-quality studies

	Surgery within 6h		Surgery after 6h			Odds Ratio
Study or Subgroup	Events	Total	Events	Total	Weight	M-H, Random, 95% CI
Boyer et al, 2009	8	12	16	21	12.2%	0.63 [0.13, 2.99]
Chao et al, 2013	4	27	31	94	22.8%	0.35 [0.11, 1.11]
Corona et al, 2016	2	9	4	11	0.0%	0.50 [0.07, 3.67]
Hadeed et al, 2016	3	40	8	47	15.2%	0.40 [0.10, 1.60]
Huang et al, 2008	0	11	3	5		Not estimable
Kaiser et al, 1981	3	13	5	7	0.0%	0.12 [0.01, 0.97]
Latifi et al, 2018	4	34	15	81	21.3%	0.59 [0.18, 1.92]
Nawijn et al, Aug 2019	2	20	1	5	4.3%	0.44 [0.03, 6.19]
Nawijn et al, Feb 2019	8	30	12	27	24.3%	0.45 [0.15, 1.38]
Tsai et al, 2015	5	11	3	7		Not estimable
**Total (95% CI)**		**163**		**275**	**100.0%**	**0.46 [0.27, 0.80]**
Total events	29		83			

Heterogeneity: Tau² = 0.00; Chi² = 0.56, df = 5 (P = 0.99); I² = 0%
Test for overall effect: Z = 2.78 (P = 0.005)

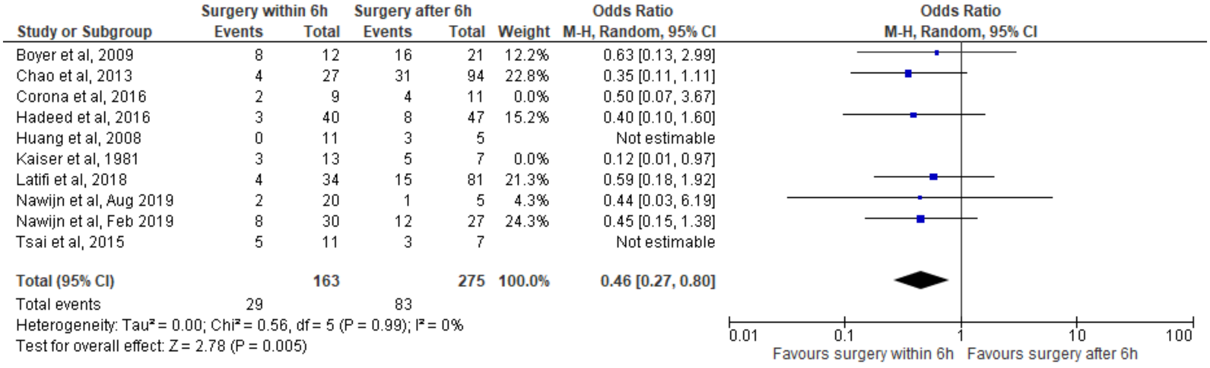

Subgroup analysis: Studies published ≥2009

	Surgery within 6h		Surgery after 6h			Odds Ratio
Study or Subgroup	Events	Total	Events	Total	Weight	M-H, Random, 95% CI
Boyer et al, 2009	8	12	16	21	10.5%	0.63 [0.13, 2.99]
Chao et al, 2013	4	27	31	94	19.7%	0.35 [0.11, 1.11]
Corona et al, 2016	2	9	4	11	6.5%	0.50 [0.07, 3.67]
Hadeed et al, 2016	3	40	8	47	13.1%	0.40 [0.10, 1.60]
Huang et al, 2008	0	11	3	5	0.0%	0.03 [0.00, 0.81]
Kaiser et al, 1981	3	13	5	7	0.0%	0.12 [0.01, 0.97]
Latifi et al, 2018	4	34	15	81	18.4%	0.59 [0.18, 1.92]
Nawijn et al, Aug 2019	2	20	1	5	3.7%	0.44 [0.03, 6.19]
Nawijn et al, Feb 2019	8	30	12	27	21.0%	0.45 [0.15, 1.38]
Tsai et al, 2015	5	11	3	7	7.1%	1.11 [0.16, 7.51]
**Total (95% CI)**		**183**		**293**	**100.0%**	**0.49 [0.30, 0.82]**
Total events	36		90			

Heterogeneity: Tau² = 0.00; Chi² = 1.31, df = 7 (P = 0.99); I² = 0%
Test for overall effect: Z = 2.73 (P = 0.006)

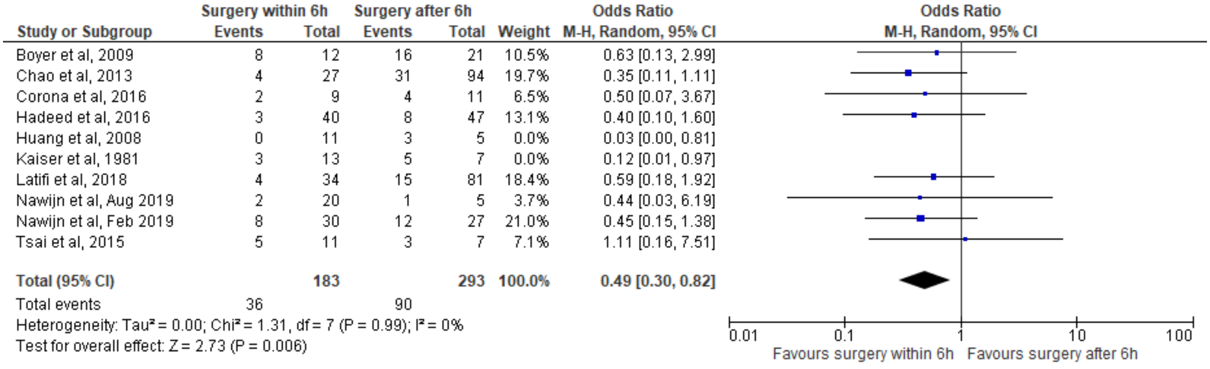

Subgroup analysis: Studies without limitation on affected body region by NSTI

	Surgery within 6h		Surgery after 6h			Odds Ratio
**Study or Subgroup**	**Events**	**Total**	**Events**	**Total**	**Weight**	**M-H, Random, 95% CI**
Boyer et al, 2009	8	12	16	21	0.0%	0.63 [0.13, 2.99]
Chao et al, 2013	4	27	31	94	22.1%	0.35 [0.11, 1.11]
Corona et al, 2016	2	9	4	11	0.0%	0.50 [0.07, 3.67]
Hadeed et al, 2016	3	40	8	47	14.8%	0.40 [0.10, 1.60]
Huang et al, 2008	0	11	3	5	0.0%	0.03 [0.00, 0.81]
Kaiser et al, 1981	3	13	5	7	6.7%	0.12 [0.01, 0.97]
Latifi et al, 2018	4	34	15	81	20.7%	0.59 [0.18, 1.92]
Nawijn et al, Aug 2019	2	20	1	5	4.2%	0.44 [0.03, 6.19]
Nawijn et al, Feb 2019	8	30	12	27	23.6%	0.45 [0.15, 1.38]
Tsai et al, 2015	5	11	3	7	8.0%	1.11 [0.16, 7.51]
**Total (95% CI)**		**175**		**268**	**100.0%**	**0.44 [0.25, 0.75]**
Total events	29		75			

Heterogeneity: Tau² = 0.00; Chi² = 2.78, df = 6 (P = 0.84); I² = 0%

Test for overall effect: Z = 3.02 (P = 0.003)

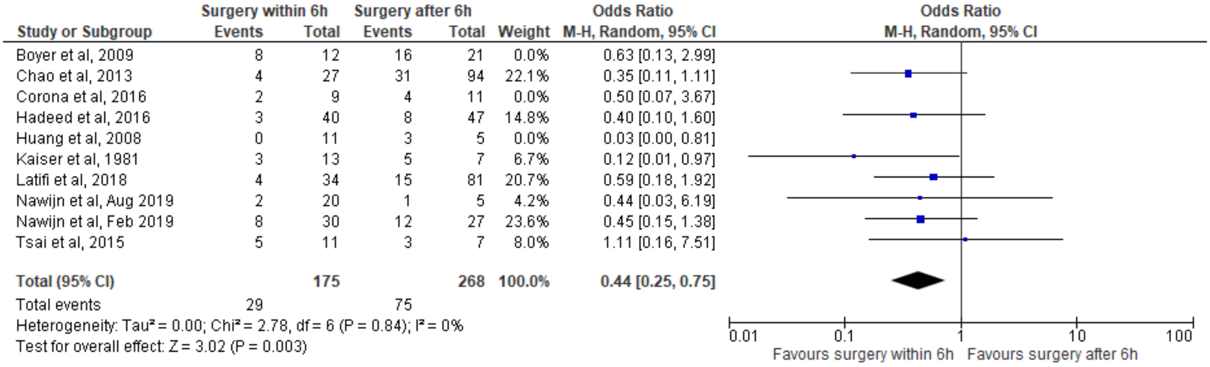

Subgroup analysis: Excluding studies limited to specific microbial types of NSTI

	Surgery within 6h		Surgery after 6h			Odds Ratio
**Study or Subgroup**	**Events**	**Total**	**Events**	**Total**	**Weight**	**M-H, Random, 95% CI**
Boyer et al, 2009	8	12	16	21	13.3%	0.63 [0.13, 2.99]
Chao et al, 2013	4	27	31	94	0.0%	0.35 [0.11, 1.11]
Corona et al, 2016	2	9	4	11	8.2%	0.50 [0.07, 3.67]
Hadeed et al, 2016	3	40	8	47	16.6%	0.40 [0.10, 1.60]
Huang et al, 2008	0	11	3	5	0.0%	0.03 [0.00, 0.81]
Kaiser et al, 1981	3	13	5	7	7.5%	0.12 [0.01, 0.97]
Latifi et al, 2018	4	34	15	81	23.2%	0.59 [0.18, 1.92]
Nawijn et al, Aug 2019	2	20	1	5	4.7%	0.44 [0.03, 6.19]
Nawijn et al, Feb 2019	8	30	12	27	26.5%	0.45 [0.15, 1.38]
Tsai et al, 2015	5	11	3	7	0.0%	1.11 [0.16, 7.51]
**Total (95% CI)**		**158**		**199**	**100.0%**	**0.45 [0.25, 0.79]**
Total events	30		61			

Heterogeneity: Tau² = 0.00; Chi² = 1.95, df = 6 (P = 0.92); I² = 0%

Test for overall effect: Z = 2.76 (P = 0.006)

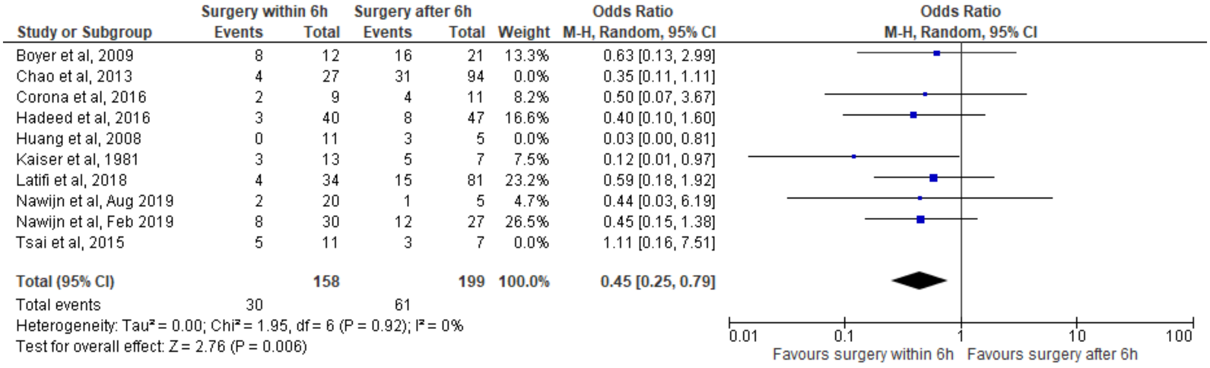

Surgery within 6 hours after presentation - amputation as outcome

Study or Subgroup	Surgery within 6h Events	Surgery within 6h Total	Surgery after 6h Events	Surgery after 6h Total	Weight	Odds Ratio M-H, Random, 95% CI
Corona et al, 2016	1	9	3	11	8.4%	0.33 [0.03, 3.93]
Hadeed et al, 2016	6	29	13	31	38.6%	0.36 [0.11, 1.14]
Huang et al, 2008	4	11	0	5	5.2%	6.60 [0.29, 149.77]
Kaiser et al, 1981	0	7	1	3	4.1%	0.11 [0.00, 3.70]
Mittapalli et al, 2015	2	6	3	18	11.5%	2.50 [0.31, 20.45]
Nawijn et al, Aug 2019	1	14	0	4	4.5%	1.00 [0.03, 29.19]
Nawijn et al, Feb 2019	4	16	3	15	17.6%	1.33 [0.24, 7.28]
Tsai et al, 2015	2	11	2	7	10.1%	0.56 [0.06, 5.24]
**Total (95% CI)**		**103**		**94**	**100.0%**	**0.68 [0.34, 1.39]**
Total events	20		25			

Heterogeneity: Tau² = 0.00; Chi² = 6.72, df = 7 (P = 0.46); I² = 0%
Test for overall effect: Z = 1.05 (P = 0.30)

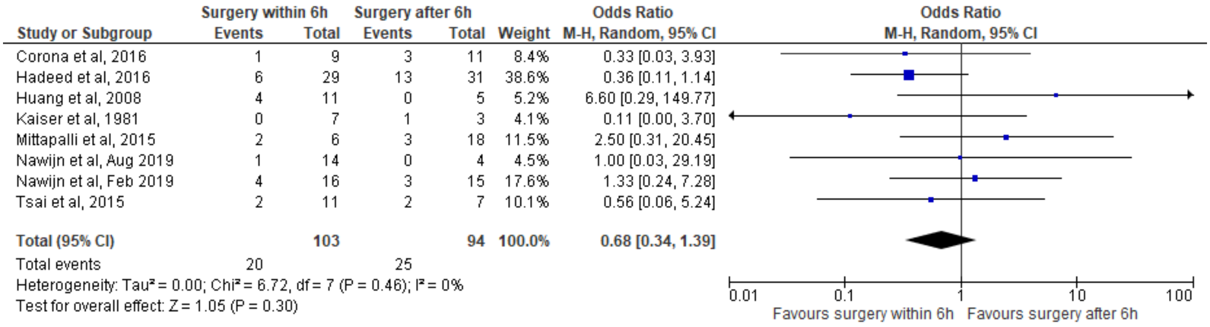

Subgroup analysis: High-quality studies

Study or Subgroup	Surgery within 6h Events	Surgery within 6h Total	Surgery after 6h Events	Surgery after 6h Total	Weight	Odds Ratio M-H, Random, 95% CI
Corona et al, 2016	1	9	3	11	0.0%	0.33 [0.03, 3.93]
Hadeed et al, 2016	6	29	13	31	63.6%	0.36 [0.11, 1.14]
Huang et al, 2008	4	11	0	5		Not estimable
Kaiser et al, 1981	0	7	1	3	0.0%	0.11 [0.00, 3.70]
Mittapalli et al, 2015	2	6	3	18	0.0%	2.50 [0.31, 20.45]
Nawijn et al, Aug 2019	1	14	0	4	7.4%	1.00 [0.03, 29.19]
Nawijn et al, Feb 2019	4	16	3	15	29.1%	1.33 [0.24, 7.28]
Tsai et al, 2015	2	11	2	7		Not estimable
**Total (95% CI)**		**59**		**50**	**100.0%**	**0.57 [0.23, 1.42]**
Total events	11		16			

Heterogeneity: Tau² = 0.00; Chi² = 1.68, df = 2 (P = 0.43); I² = 0%
Test for overall effect: Z = 1.21 (P = 0.23)

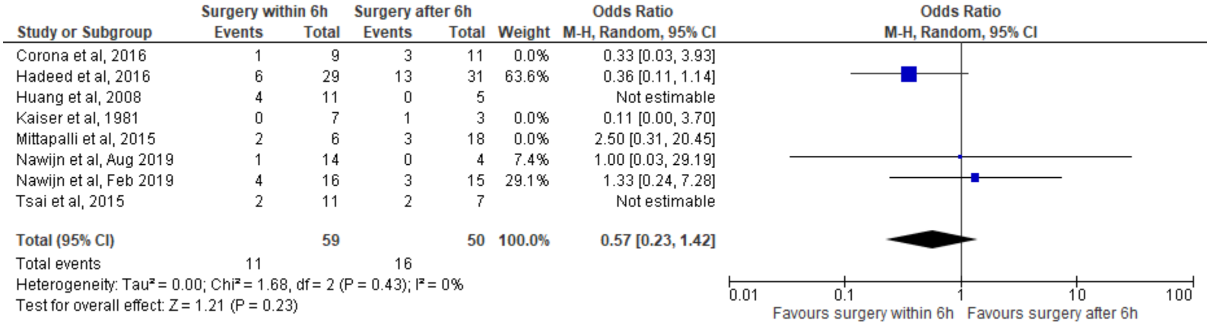

Subgroup analysis: Studies published ≥2009

Study or Subgroup	Surgery within 6h Events	Surgery within 6h Total	Surgery after 6h Events	Surgery after 6h Total	Weight	Odds Ratio M-H, Random, 95% CI
Corona et al, 2016	1	9	3	11	9.2%	0.33 [0.03, 3.93]
Hadeed et al, 2016	6	29	13	31	42.6%	0.36 [0.11, 1.14]
Huang et al, 2008	4	11	0	5	0.0%	6.60 [0.29, 149.77]
Kaiser et al, 1981	0	7	1	3	0.0%	0.11 [0.00, 3.70]
Mittapalli et al, 2015	2	6	3	18	12.7%	2.50 [0.31, 20.45]
Nawijn et al, Aug 2019	1	14	0	4	4.9%	1.00 [0.03, 29.19]
Nawijn et al, Feb 2019	4	16	3	15	19.5%	1.33 [0.24, 7.28]
Tsai et al, 2015	2	11	2	7	11.1%	0.56 [0.06, 5.24]
**Total (95% CI)**		**85**		**86**	**100.0%**	**0.65 [0.31, 1.38]**
Total events	16		24			

Heterogeneity: Tau² = 0.00; Chi² = 3.64, df = 5 (P = 0.60); I² = 0%
Test for overall effect: Z = 1.12 (P = 0.26)

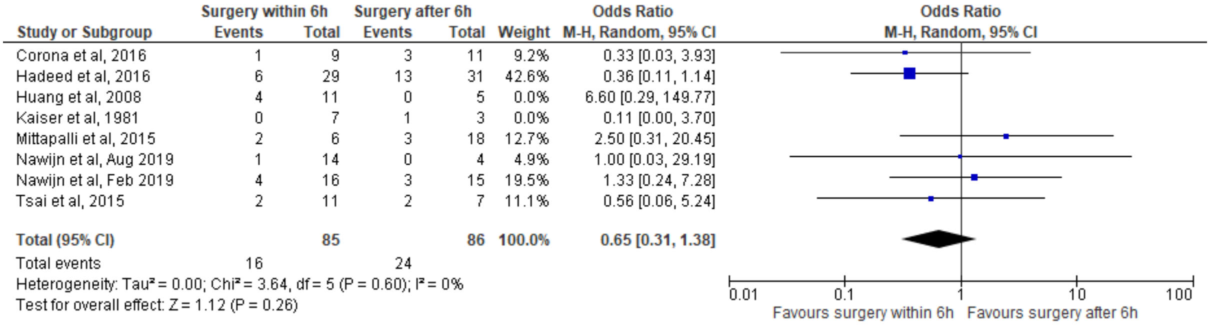

Subgroup analysis: Studies without limitation on affected body region by NSTI

Study or Subgroup	Surgery within 6h Events	Surgery within 6h Total	Surgery after 6h Events	Surgery after 6h Total	Weight	Odds Ratio M-H, Random, 95% CI
Corona et al, 2016	1	9	3	11	0.0%	0.33 [0.03, 3.93]
Hadeed et al, 2016	6	29	13	31	44.7%	0.36 [0.11, 1.14]
Huang et al, 2008	4	11	0	5	0.0%	6.60 [0.29, 149.77]
Kaiser et al, 1981	0	7	1	3	4.8%	0.11 [0.00, 3.70]
Mittapalli et al, 2015	2	6	3	18	13.3%	2.50 [0.31, 20.45]
Nawijn et al, Aug 2019	1	14	0	4	5.2%	1.00 [0.03, 29.19]
Nawijn et al, Feb 2019	4	16	3	15	20.4%	1.33 [0.24, 7.28]
Tsai et al, 2015	2	11	2	7	11.7%	0.56 [0.06, 5.24]
**Total (95% CI)**		**83**		**78**	**100.0%**	**0.64 [0.30, 1.38]**
Total events	15		22			

Heterogeneity: Tau² = 0.00; Chi² = 4.33, df = 5 (P = 0.50); I² = 0%
Test for overall effect: Z = 1.14 (P = 0.25)

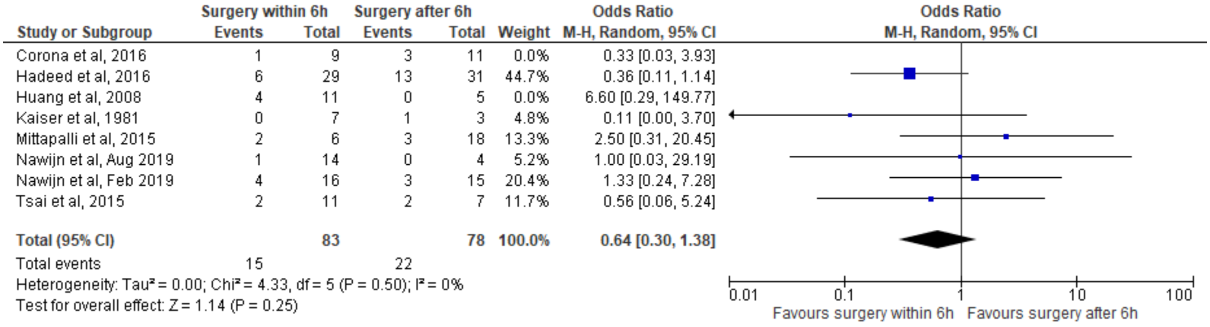

Subgroup analysis: Excluding studies limited to specific microbial types of NSTI

	Surgery within 6h		Surgery after 6h			Odds Ratio	Odds Ratio
**Study or Subgroup**	**Events**	**Total**	**Events**	**Total**	**Weight**	**M-H, Random, 95% CI**	**M-H, Random, 95% CI**
Corona et al, 2016	1	9	3	11	9.9%	0.33 [0.03, 3.93]	
Hadeed et al, 2016	6	29	13	31	45.6%	0.36 [0.11, 1.14]	
Huang et al, 2008	4	11	0	5	0.0%	6.60 [0.29, 149.77]	
Kaiser et al, 1981	0	7	1	3	4.9%	0.11 [0.00, 3.70]	
Mittapalli et al, 2015	2	6	3	18	13.6%	2.50 [0.31, 20.45]	
Nawijn et al, Aug 2019	1	14	0	4	5.3%	1.00 [0.03, 29.19]	
Nawijn et al, Feb 2019	4	16	3	15	20.8%	1.33 [0.24, 7.28]	
Tsai et al, 2015	2	11	2	7	0.0%	0.56 [0.06, 5.24]	
**Total (95% CI)**		**81**		**82**	**100.0%**	**0.61 [0.28, 1.32]**	
Total events	14		23				

Heterogeneity: Tau² = 0.00; Chi² = 4.57, df = 5 (P = 0.47); I² = 0%

Test for overall effect: Z = 1.25 (P = 0.21)

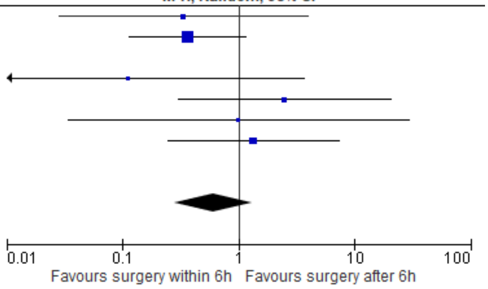

Surgery within 12 hours after presentation - mortality as outcome

Study or Subgroup	Surgery within 12h Events	Surgery within 12h Total	Surgery after 12h Events	Surgery after 12h Total	Weight	Odds Ratio M-H, Random, 95% CI
Balci et al, 2009	0	6	2	5	1.6%	0.11 [0.00, 2.93]
Boyer et al, 2009	9	16	15	17	5.4%	0.17 [0.03, 1.01]
Chao et al, 2013	8	53	27	68	21.3%	0.27 [0.11, 0.66]
Corona et al, 2016	3	14	3	6	4.1%	0.27 [0.04, 2.11]
Huang et al, 2008	2	13	1	3	2.1%	0.36 [0.02, 6.19]
Kaiser et al, 1981	4	15	4	5	2.8%	0.09 [0.01, 1.08]
Kobayashi et al, 2011	1	22	7	25	3.6%	0.12 [0.01, 1.09]
Lee et al, 2014	10	67	8	33	15.7%	0.55 [0.19, 1.55]
Lille et al, 1996	1	17	3	12	3.0%	0.19 [0.02, 2.08]
Nawijn et al, Aug 2019	3	24	0	2	1.6%	0.81 [0.03, 20.78]
Nawijn et al, Feb 2019	12	42	9	28	15.9%	0.84 [0.30, 2.38]
Pakula et al, 2012	5	35	3	19	7.1%	0.89 [0.19, 4.21]
Sudarsky et al, 1987	0	7	2	26	1.7%	0.65 [0.03, 15.16]
Tsai et al, 2010	12	54	6	17	12.2%	0.52 [0.16, 1.71]
Tsai et al, 2015	7	16	1	2	2.0%	0.78 [0.04, 14.75]
**Total (95% CI)**		**401**		**268**	**100.0%**	**0.41 [0.27, 0.61]**
Total events	77		91			

Heterogeneity: Tau² = 0.00; Chi² = 9.31, df = 14 (P = 0.81); I² = 0%
Test for overall effect: Z = 4.27 (P < 0.0001)

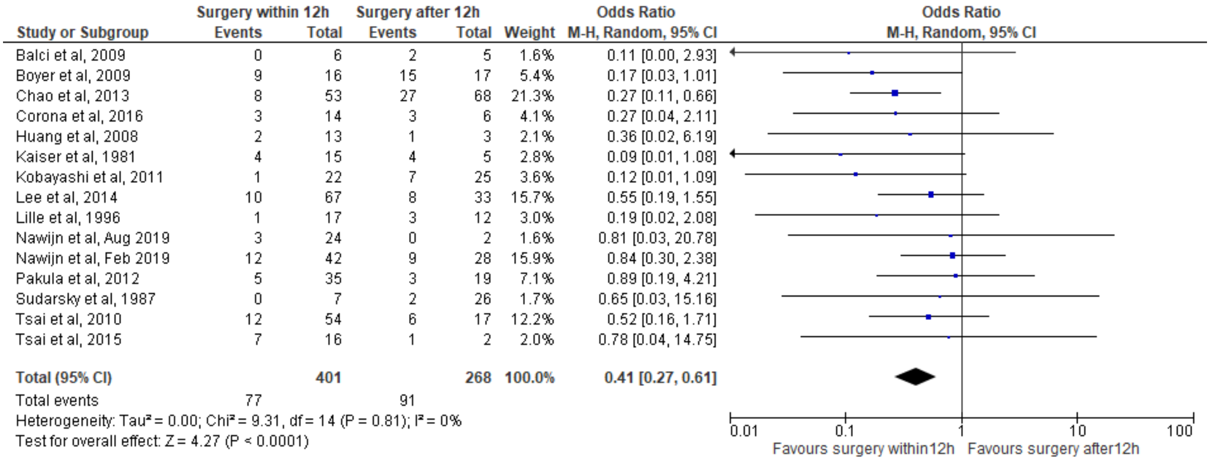

Subgroup analysis: High-quality studies

Study or Subgroup	Surgery within 12h Events	Surgery within 12h Total	Surgery after 12h Events	Surgery after 12h Total	Weight	Odds Ratio M-H, Random, 95% CI
Balci et al, 2009	0	6	2	5	0.0%	0.11 [0.00, 2.93]
Boyer et al, 2009	9	16	15	17	9.1%	0.17 [0.03, 1.01]
Chao et al, 2013	8	53	27	68	32.5%	0.27 [0.11, 0.66]
Corona et al, 2016	3	14	3	6	0.0%	0.27 [0.04, 2.11]
Huang et al, 2008	2	13	1	3		Not estimable
Kaiser et al, 1981	4	15	4	5	0.0%	0.09 [0.01, 1.08]
Kobayashi et al, 2011	1	22	7	25	6.0%	0.12 [0.01, 1.09]
Lee et al, 2014	10	67	8	33	24.7%	0.55 [0.19, 1.55]
Lille et al, 1996	1	17	3	12	0.0%	0.19 [0.02, 2.08]
Nawijn et al, Aug 2019	3	24	0	2	2.8%	0.81 [0.03, 20.78]
Nawijn et al, Feb 2019	12	42	9	28	24.9%	0.84 [0.30, 2.38]
Pakula et al, 2012	5	35	3	19	0.0%	0.89 [0.19, 4.21]
Sudarsky et al, 1987	0	7	2	26	0.0%	0.65 [0.03, 15.16]
Tsai et al, 2010	12	54	6	17		Not estimable
Tsai et al, 2015	7	16	1	2		Not estimable
**Total (95% CI)**		**224**		**173**	**100.0%**	**0.40 [0.23, 0.69]**
Total events	43		66			

Heterogeneity: Tau² = 0.03; Chi² = 5.30, df = 5 (P = 0.38); I² = 6%
Test for overall effect: Z = 3.28 (P = 0.001)

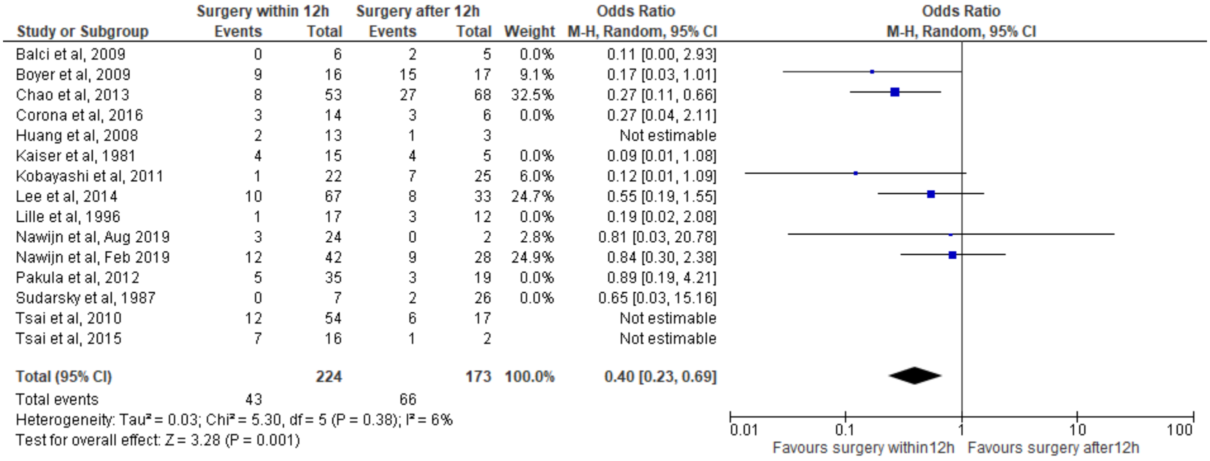

Subgroup analysis: Studies published ≥2009

Study or Subgroup	Surgery within 12h Events	Surgery within 12h Total	Surgery after 12h Events	Surgery after 12h Total	Weight	Odds Ratio M-H, Random, 95% CI
Balci et al, 2009	0	6	2	5	1.7%	0.11 [0.00, 2.93]
Boyer et al, 2009	9	16	15	17	6.0%	0.17 [0.03, 1.01]
Chao et al, 2013	8	53	27	68	23.6%	0.27 [0.11, 0.66]
Corona et al, 2016	3	14	3	6	4.5%	0.27 [0.04, 2.11]
Huang et al, 2008	2	13	1	3	0.0%	0.36 [0.02, 6.19]
Kaiser et al, 1981	4	15	4	5	0.0%	0.09 [0.01, 1.08]
Kobayashi et al, 2011	1	22	7	25	3.9%	0.12 [0.01, 1.09]
Lee et al, 2014	10	67	8	33	17.4%	0.55 [0.19, 1.55]
Lille et al, 1996	1	17	3	12	0.0%	0.19 [0.02, 2.08]
Nawijn et al, Aug 2019	3	24	0	2	1.8%	0.81 [0.03, 20.78]
Nawijn et al, Feb 2019	12	42	9	28	17.5%	0.84 [0.30, 2.38]
Pakula et al, 2012	5	35	3	19	7.8%	0.89 [0.19, 4.21]
Sudarsky et al, 1987	0	7	2	26	0.0%	0.65 [0.03, 15.16]
Tsai et al, 2010	12	54	6	17	13.5%	0.52 [0.16, 1.71]
Tsai et al, 2015	7	16	1	2	2.2%	0.78 [0.04, 14.75]
**Total (95% CI)**		**349**		**222**	**100.0%**	**0.43 [0.28, 0.67]**
Total events	70		81			

Heterogeneity: Tau² = 0.00; Chi² = 7.32, df = 10 (P = 0.69); I² = 0%
Test for overall effect: Z = 3.76 (P = 0.0002)

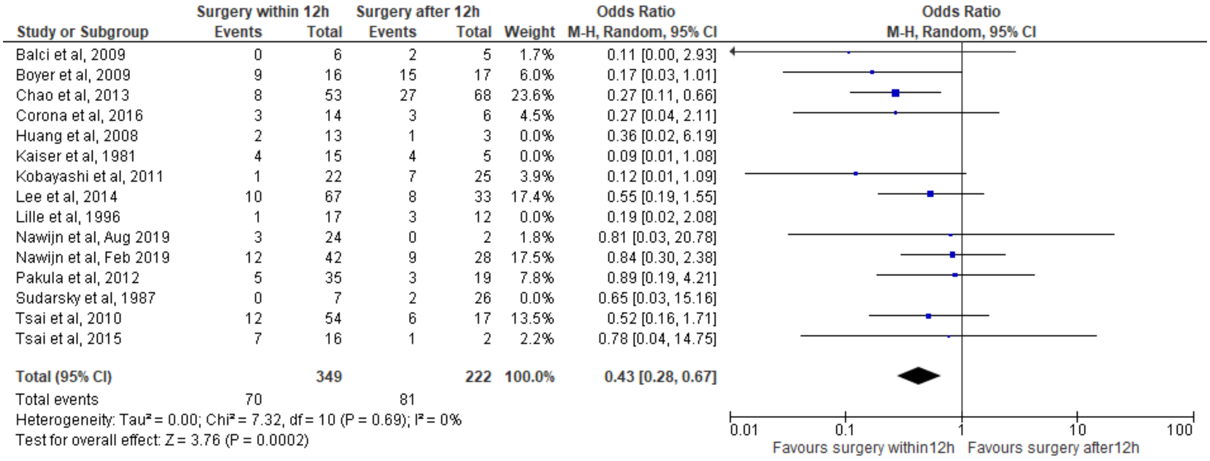

Subgroup analysis: Studies without limitation on affected body region by NSTI

Study or Subgroup	Surgery within 12h Events	Surgery within 12h Total	Surgery after 12h Events	Surgery after 12h Total	Weight	Odds Ratio M-H, Random, 95% CI
Balci et al, 2009	0	6	2	5	0.0%	0.11 [0.00, 2.93]
Boyer et al, 2009	9	16	15	17	0.0%	0.17 [0.03, 1.01]
Chao et al, 2013	8	53	27	68	24.5%	0.27 [0.11, 0.66]
Corona et al, 2016	3	14	3	6	0.0%	0.27 [0.04, 2.11]
Huang et al, 2008	2	13	1	3	0.0%	0.36 [0.02, 6.19]
Kaiser et al, 1981	4	15	4	5	3.2%	0.09 [0.01, 1.08]
Kobayashi et al, 2011	1	22	7	25	4.1%	0.12 [0.01, 1.09]
Lee et al, 2014	10	67	8	33	18.1%	0.55 [0.19, 1.55]
Lille et al, 1996	1	17	3	12	3.4%	0.19 [0.02, 2.08]
Nawijn et al, Aug 2019	3	24	0	2	1.9%	0.81 [0.03, 20.78]
Nawijn et al, Feb 2019	12	42	9	28	18.3%	0.84 [0.30, 2.38]
Pakula et al, 2012	5	35	3	19	8.1%	0.89 [0.19, 4.21]
Sudarsky et al, 1987	0	7	2	26	2.0%	0.65 [0.03, 15.16]
Tsai et al, 2010	12	54	6	17	14.0%	0.52 [0.16, 1.71]
Tsai et al, 2015	7	16	1	2	2.3%	0.78 [0.04, 14.75]
**Total (95% CI)**		**352**		**237**	**100.0%**	**0.45 [0.29, 0.70]**
Total events	63		70			

Heterogeneity: Tau² = 0.00; Chi² = 7.44, df = 10 (P = 0.68); I² = 0%
Test for overall effect: Z = 3.54 (P = 0.0004)

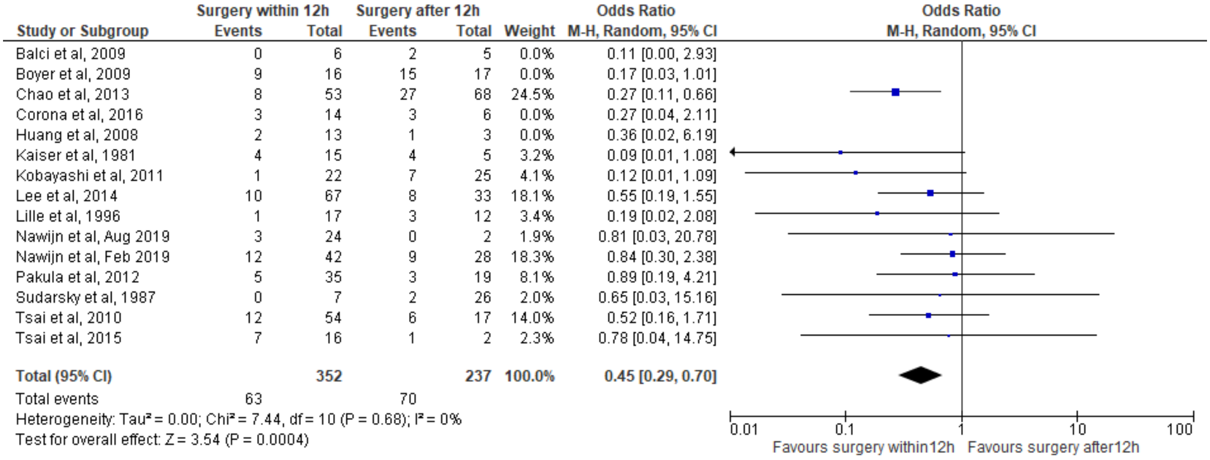

Subgroup analysis: Excluding studies limited to specific microbial types of NSTI

Study or Subgroup	Surgery within 12h Events	Surgery within 12h Total	Surgery after 12h Events	Surgery after 12h Total	Weight	Odds Ratio M-H, Random, 95% CI
Balci et al, 2009	0	6	2	5	3.4%	0.11 [0.00, 2.93]
Boyer et al, 2009	9	16	15	17	11.6%	0.17 [0.03, 1.01]
Chao et al, 2013	8	53	27	68	0.0%	0.27 [0.11, 0.66]
Corona et al, 2016	3	14	3	6	8.7%	0.27 [0.04, 2.11]
Huang et al, 2008	2	13	1	3	0.0%	0.36 [0.02, 6.19]
Kaiser et al, 1981	4	15	4	5	6.0%	0.09 [0.01, 1.08]
Kobayashi et al, 2011	1	22	7	25	7.6%	0.12 [0.01, 1.09]
Lee et al, 2014	10	67	8	33	0.0%	0.55 [0.19, 1.55]
Lille et al, 1996	1	17	3	12	6.3%	0.19 [0.02, 2.08]
Nawijn et al, Aug 2019	3	24	0	2	3.5%	0.81 [0.03, 20.78]
Nawijn et al, Feb 2019	12	42	9	28	34.0%	0.84 [0.30, 2.38]
Pakula et al, 2012	5	35	3	19	15.1%	0.89 [0.19, 4.21]
Sudarsky et al, 1987	0	7	2	26	3.7%	0.65 [0.03, 15.16]
Tsai et al, 2010	12	54	6	17	0.0%	0.52 [0.16, 1.71]
Tsai et al, 2015	7	16	1	2	0.0%	0.78 [0.04, 14.75]
**Total (95% CI)**		**198**		**145**	**100.0%**	**0.41 [0.22, 0.74]**
Total events	38		48			

Heterogeneity: Tau² = 0.00; Chi² = 7.83, df = 9 (P = 0.55); I² = 0%
Test for overall effect: Z = 2.92 (P = 0.003)

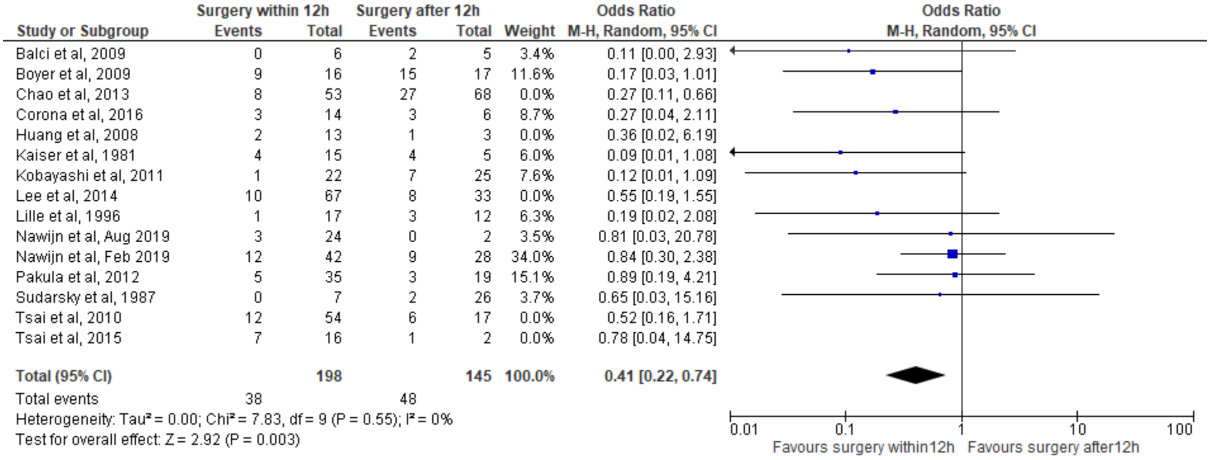

Surgery within 12 hours after presentation - amputation as outcome

Study or Subgroup	Surgery within 12h Events	Surgery within 12h Total	Surgery after 12h Events	Surgery after 12h Total	Weight	Odds Ratio M-H, Random, 95% CI
Corona et al, 2016	2	14	2	6	17.3%	0.33 [0.03, 3.20]
Huang et al, 2008	4	13	0	3	8.8%	3.32 [0.14, 78.81]
Kaiser et al, 1981	0	7	1	3	7.2%	0.11 [0.00, 3.70]
Mittapalli et al, 2015	2	8	3	16	21.4%	1.44 [0.19, 11.04]
Nawijn et al, Aug 2019	1	17	0	1	6.8%	0.27 [0.01, 10.09]
Nawijn et al, Feb 2019	4	21	3	11	30.0%	0.63 [0.11, 3.49]
Tsai et al, 2015	4	16	0	2	8.5%	1.80 [0.07, 45.14]
**Total (95% CI)**		**96**		**42**	**100.0%**	**0.71 [0.28, 1.82]**
Total events	17		9			

Heterogeneity: Tau² = 0.00; Chi² = 3.51, df = 6 (P = 0.74); I² = 0%
Test for overall effect: Z = 0.71 (P = 0.48)

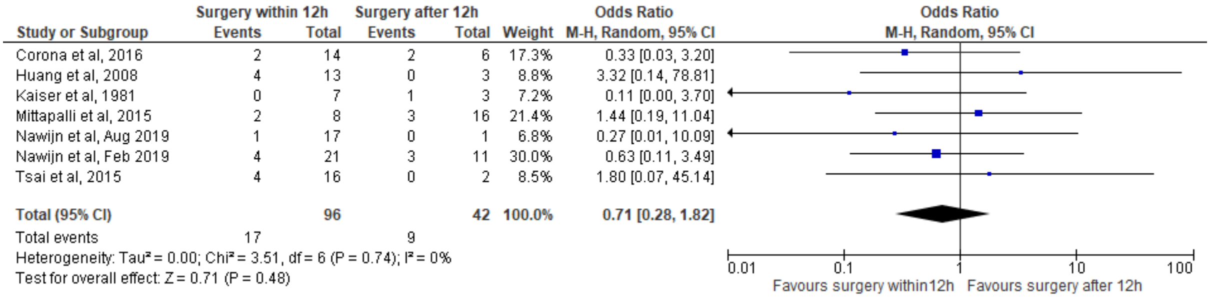

Subgroup analysis: High-quality studies

Study or Subgroup	Surgery within 12h Events	Surgery within 12h Total	Surgery after 12h Events	Surgery after 12h Total	Weight	Odds Ratio M-H, Random, 95% CI
Corona et al, 2016	2	14	2	6	0.0%	0.33 [0.03, 3.20]
Huang et al, 2008	4	13	0	3		Not estimable
Kaiser et al, 1981	0	7	1	3	0.0%	0.11 [0.00, 3.70]
Mittapalli et al, 2015	2	8	3	16	0.0%	1.44 [0.19, 11.04]
Nawijn et al, Aug 2019	1	17	0	1	18.4%	0.27 [0.01, 10.09]
Nawijn et al, Feb 2019	4	21	3	11	81.6%	0.63 [0.11, 3.49]
Tsai et al, 2015	4	16	0	2		Not estimable
**Total (95% CI)**		**38**		**12**	**100.0%**	**0.54 [0.11, 2.54]**
Total events	5		3			

Heterogeneity: Tau² = 0.00; Chi² = 0.17, df = 1 (P = 0.68); I² = 0%
Test for overall effect: Z = 0.78 (P = 0.43)

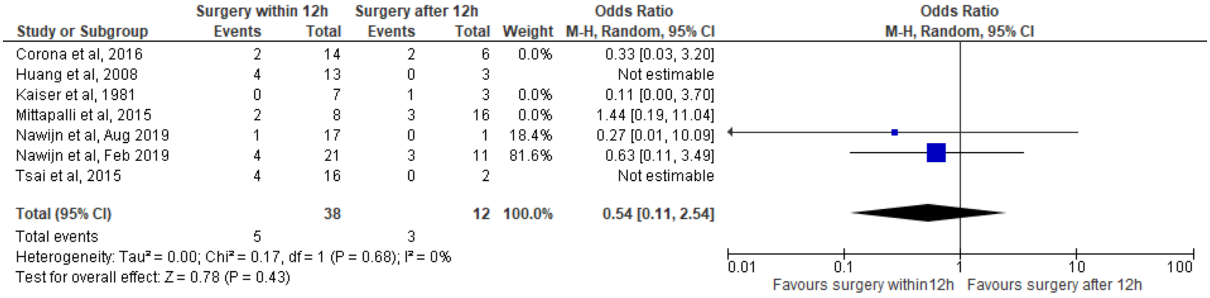

Subgroup analysis: Studies published ≥2009

Study or Subgroup	Surgery within 12h Events	Surgery within 12h Total	Surgery after 12h Events	Surgery after 12h Total	Weight	Odds Ratio M-H, Random, 95% CI
Corona et al, 2016	2	14	2	6	20.6%	0.33 [0.03, 3.20]
Huang et al, 2008	4	13	0	3	0.0%	3.32 [0.14, 78.81]
Kaiser et al, 1981	0	7	1	3	0.0%	0.11 [0.00, 3.70]
Mittapalli et al, 2015	2	8	3	16	25.5%	1.44 [0.19, 11.04]
Nawijn et al, Aug 2019	1	17	0	1	8.1%	0.27 [0.01, 10.09]
Nawijn et al, Feb 2019	4	21	3	11	35.7%	0.63 [0.11, 3.49]
Tsai et al, 2015	4	16	0	2	10.1%	1.80 [0.07, 45.14]
**Total (95% CI)**		**76**		**36**	**100.0%**	**0.71 [0.25, 1.98]**
Total events	13		8			

Heterogeneity: Tau² = 0.00; Chi² = 1.51, df = 4 (P = 0.82); I² = 0%
Test for overall effect: Z = 0.66 (P = 0.51)

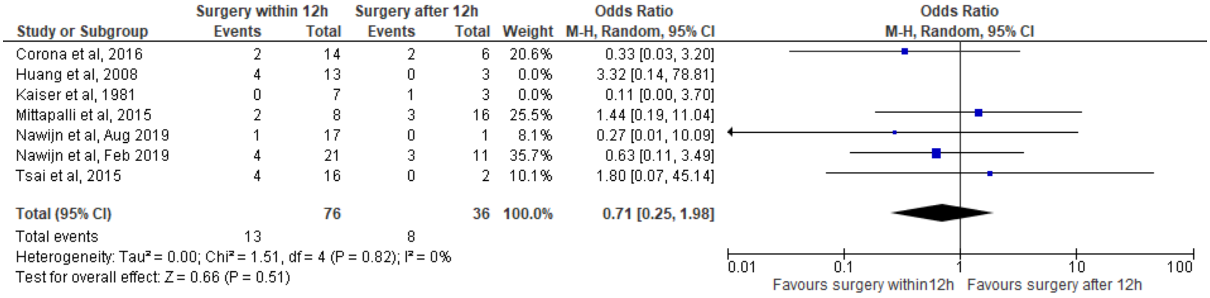

Subgroup analysis: Studies without limitation on affected body region by NSTI

Study or Subgroup	Surgery within 12h Events	Surgery within 12h Total	Surgery after 12h Events	Surgery after 12h Total	Weight	Odds Ratio M-H, Random, 95% CI
Corona et al, 2016	2	14	2	6	0.0%	0.33 [0.03, 3.20]
Huang et al, 2008	4	13	0	3	0.0%	3.32 [0.14, 78.81]
Kaiser et al, 1981	0	7	1	3	9.7%	0.11 [0.00, 3.70]
Mittapalli et al, 2015	2	8	3	16	28.9%	1.44 [0.19, 11.04]
Nawijn et al, Aug 2019	1	17	0	1	9.2%	0.27 [0.01, 10.09]
Nawijn et al, Feb 2019	4	21	3	11	40.6%	0.63 [0.11, 3.49]
Tsai et al, 2015	4	16	0	2	11.5%	1.80 [0.07, 45.14]
**Total (95% CI)**		**69**		**33**	**100.0%**	**0.71 [0.24, 2.11]**
Total events	11		7			

Heterogeneity: Tau² = 0.00; Chi² = 2.15, df = 4 (P = 0.71); I² = 0%
Test for overall effect: Z = 0.62 (P = 0.53)

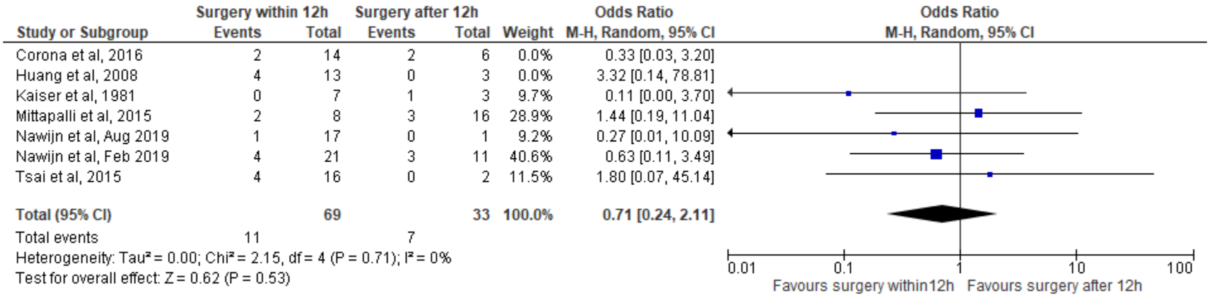

Subgroup analysis: Excluding studies limited to specific microbial types of NSTI

	Surgery within 12h		Surgery after 12h			Odds Ratio
Study or Subgroup	Events	Total	Events	Total	Weight	M-H, Random, 95% CI
Corona et al, 2016	2	14	2	6	20.9%	0.33 [0.03, 3.20]
Huang et al, 2008	4	13	0	3	0.0%	3.32 [0.14, 78.81]
Kaiser et al, 1981	0	7	1	3	8.7%	0.11 [0.00, 3.70]
Mittapalli et al, 2015	2	8	3	16	25.9%	1.44 [0.19, 11.04]
Nawijn et al, Aug 2019	1	17	0	1	8.2%	0.27 [0.01, 10.09]
Nawijn et al, Feb 2019	4	21	3	11	36.3%	0.63 [0.11, 3.49]
Tsai et al, 2015	4	16	0	2	0.0%	1.80 [0.07, 45.14]
**Total (95% CI)**		**67**		**37**	**100.0%**	**0.55 [0.19, 1.54]**
Total events	9		9			

Heterogeneity: Tau² = 0.00; Chi² = 2.02, df = 4 (P = 0.73); I² = 0%
Test for overall effect: Z = 1.14 (P = 0.25)

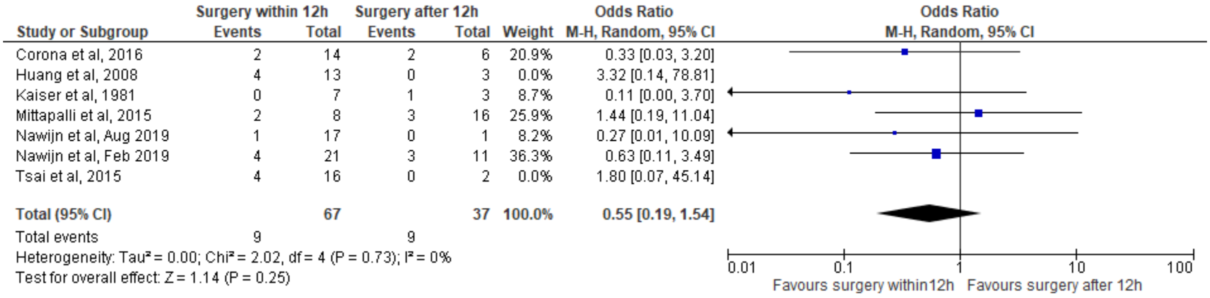

Surgery within 24 hours after presentation - mortality as outcome

Study or Subgroup	Surgery within 24h Events	Surgery within 24h Total	Surgery after 24h Events	Surgery after 24h Total	Weight	Odds Ratio M-H, Random, 95% CI
Bair et al, 2009	8	38	10	68	10.3%	1.55 [0.55, 4.33]
Balci et al, 2009	1	8	1	3	1.6%	0.29 [0.01, 6.91]
Boyer et al, 2009	23	32	1	1	1.5%	0.82 [0.03, 22.09]
Chao et al, 2013	20	90	15	31	12.7%	0.30 [0.13, 0.72]
Corona et al, 2016	6	18	0	2	1.6%	2.60 [0.11, 62.57]
George et al, 2009	8	61	0	11	1.9%	3.65 [0.20, 67.92]
Huang et al, 2008	2	14	1	2	1.6%	0.17 [0.01, 3.89]
Huang et al, 2011	38	280	19	192	18.1%	1.43 [0.80, 2.56]
Kaiser et al, 1981	6	17	2	3	2.3%	0.27 [0.02, 3.67]
Kalaivani et al, 2012	10	35	5	25	8.2%	1.60 [0.47, 5.44]
Lee et al, 2014	12	81	6	19	9.0%	0.38 [0.12, 1.18]
Nawijn et al, Feb 2019	14	51	7	20	9.4%	0.70 [0.23, 2.12]
Ogilvie et al, 2006	13	134	1	16	3.4%	1.61 [0.20, 13.21]
Palmer et al, 1995	10	21	3	9	5.3%	1.82 [0.36, 9.27]
Park et al, 2016	4	20	3	10	4.7%	0.58 [0.10, 3.33]
Stephenson et al, 1992	2	7	12	22	4.3%	0.33 [0.05, 2.10]
Wang et al, 1992	3	14	3	4	2.3%	0.09 [0.01, 1.22]
Yu et al, 2004	2	10	0	3	1.5%	2.06 [0.08, 54.80]
**Total (95% CI)**		**931**		**441**	**100.0%**	**0.79 [0.52, 1.20]**
Total events	182		89			
Heterogeneity: Tau² = 0.16; Chi² = 22.03, df = 17 (P = 0.18); I² = 23%						
Test for overall effect: Z = 1.12 (P = 0.26)						

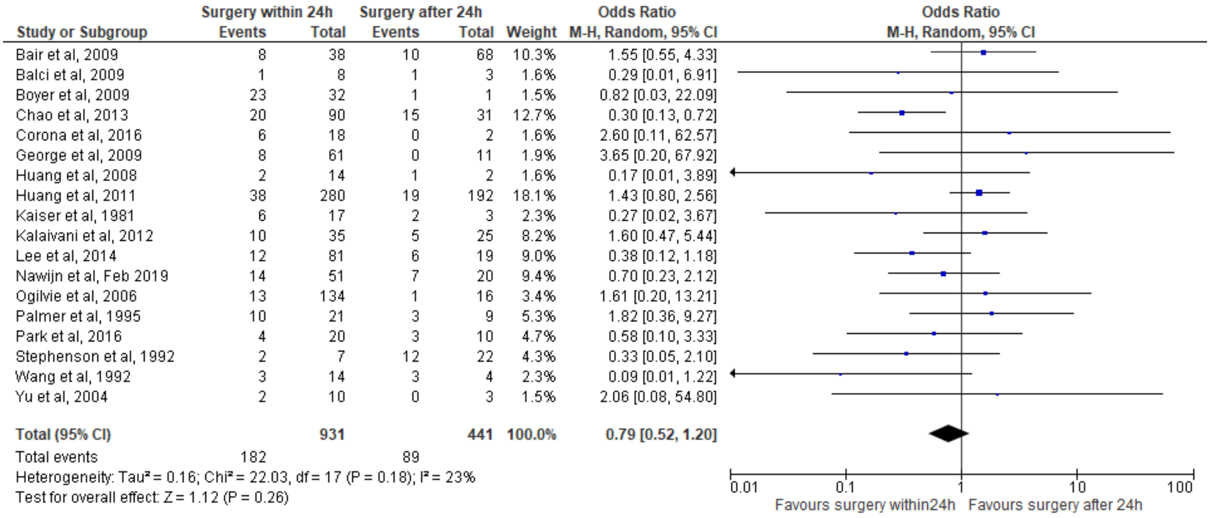

Subgroup analysis: High-quality studies

Study or Subgroup	Surgery within 24h Events	Surgery within 24h Total	Surgery after 24h Events	Surgery after 24h Total	Weight	Odds Ratio M-H, Random, 95% CI
Bair et al, 2009	8	38	10	68	0.0%	1.55 [0.55, 4.33]
Balci et al, 2009	1	8	1	3	0.0%	0.29 [0.01, 6.91]
Boyer et al, 2009	23	32	1	1	4.7%	0.82 [0.03, 22.09]
Chao et al, 2013	20	90	15	31	24.8%	0.30 [0.13, 0.72]
Corona et al, 2016	6	18	0	2	0.0%	2.60 [0.11, 62.57]
George et al, 2009	8	61	0	11	0.0%	3.65 [0.20, 67.92]
Huang et al, 2008	2	14	1	2	0.0%	0.17 [0.01, 3.89]
Huang et al, 2011	38	280	19	192	30.0%	1.43 [0.80, 2.56]
Kaiser et al, 1981	6	17	2	3	0.0%	0.27 [0.02, 3.67]
Kalaivani et al, 2012	10	35	5	25	0.0%	1.60 [0.47, 5.44]
Lee et al, 2014	12	81	6	19	19.9%	0.38 [0.12, 1.18]
Nawijn et al, Feb 2019	14	51	7	20	20.6%	0.70 [0.23, 2.12]
Ogilvie et al, 2006	13	134	1	16	0.0%	1.61 [0.20, 13.21]
Palmer et al, 1995	10	21	3	9	0.0%	1.82 [0.36, 9.27]
Park et al, 2016	4	20	3	10	0.0%	0.58 [0.10, 3.33]
Stephenson et al, 1992	2	7	12	22	0.0%	0.33 [0.05, 2.10]
Wang et al, 1992	3	14	3	4	0.0%	0.09 [0.01, 1.22]
Yu et al, 2004	2	10	0	3	0.0%	2.06 [0.08, 54.80]
**Total (95% CI)**		**534**		**263**	**100.0%**	**0.63 [0.29, 1.34]**
Total events	107		48			
Heterogeneity: Tau² = 0.41; Chi² = 10.30, df = 4 (P = 0.04); I² = 61%						
Test for overall effect: Z = 1.20 (P = 0.23)						

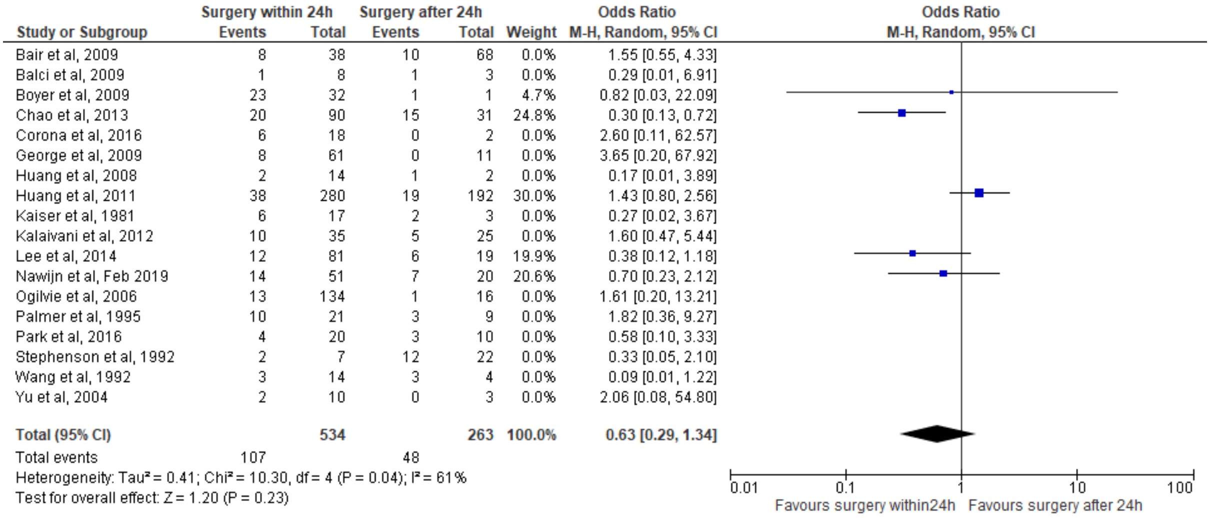

Subgroup analysis: Studies published ≥2009

Study or Subgroup	Surgery within 24h Events	Surgery within 24h Total	Surgery after 24h Events	Surgery after 24h Total	Weight	Odds Ratio M-H, Random, 95% CI
Bair et al, 2009	8	38	10	68	6.9%	1.55 [0.55, 4.33]
Balci et al, 2009	1	8	1	3	0.9%	0.29 [0.01, 6.91]
Boyer et al, 2009	23	32	1	1	0.8%	0.82 [0.03, 22.09]
Chao et al, 2013	20	90	15	31	9.0%	0.30 [0.13, 0.72]
Corona et al, 2016	6	18	0	2	0.9%	2.60 [0.11, 62.57]
George et al, 2009	8	61	0	11	1.1%	3.65 [0.20, 67.92]
Holena et al, 2011	471	4755	458	5203	30.4%	1.14 [1.00, 1.30]
Huang et al, 2008	2	14	1	2	0.0%	0.17 [0.01, 3.89]
Huang et al, 2011	38	280	19	192	14.8%	1.43 [0.80, 2.56]
Kaiser et al, 1981	6	17	2	3	0.0%	0.27 [0.02, 3.67]
Kalaivani et al, 2012	10	35	5	25	5.2%	1.60 [0.47, 5.44]
Lee et al, 2014	12	81	6	19	5.8%	0.38 [0.12, 1.18]
Nawijn et al, Feb 2019	14	51	7	20	6.1%	0.70 [0.23, 2.12]
Ogilvie et al, 2006	13	134	1	16	0.0%	1.61 [0.20, 13.21]
Palmer et al, 1995	10	21	3	9	0.0%	1.82 [0.36, 9.27]
Park et al, 2016	4	20	3	10	2.8%	0.58 [0.10, 3.33]
Stephenson et al, 1992	2	7	12	22	0.0%	0.33 [0.05, 2.10]
Sugihara et al, 2012	42	262	23	117	15.4%	0.78 [0.44, 1.37]
Wang et al, 1992	3	14	3	4	0.0%	0.09 [0.01, 1.22]
Yu et al, 2004	2	10	0	3	0.0%	2.06 [0.08, 54.80]
**Total (95% CI)**		**5731**		**5702**	**100.0%**	**0.92 [0.68, 1.25]**
Total events	657		548			
Heterogeneity: Tau² = 0.07; Chi² = 17.87, df = 12 (P = 0.12); I² = 33%						
Test for overall effect: Z = 0.53 (P = 0.60)						

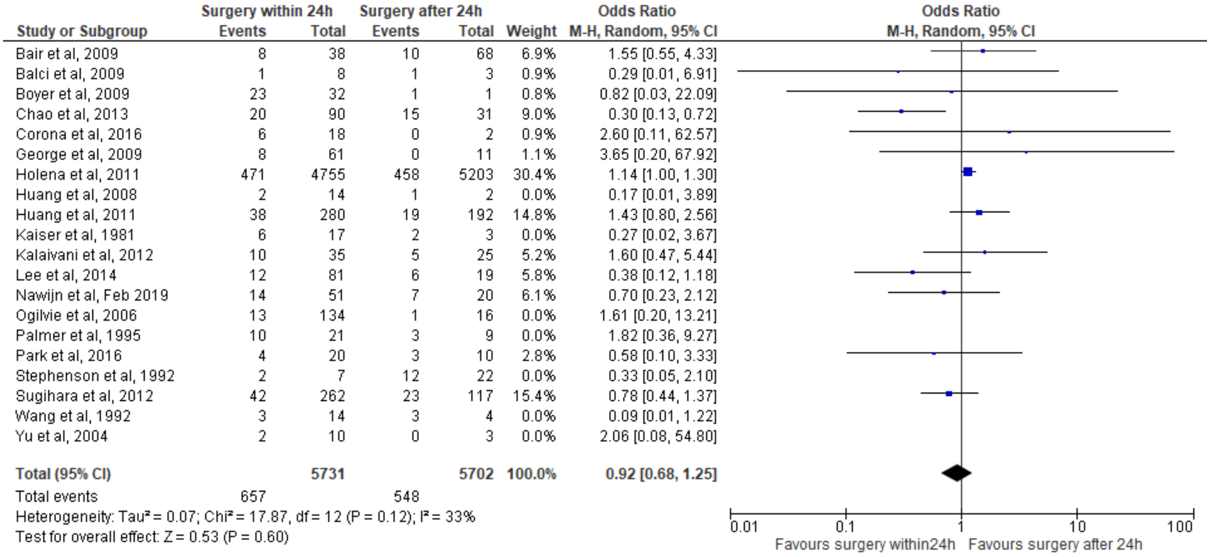

Subgroup analysis: Studies without limitation on affected body region by NSTI

Study or Subgroup	Surgery within 24h Events	Surgery within 24h Total	Surgery after 24h Events	Surgery after 24h Total	Weight	Odds Ratio M-H, Random, 95% CI
Bair et al, 2009	8	38	10	68	12.7%	1.55 [0.55, 4.33]
Balci et al, 2009	1	8	1	3	0.0%	0.29 [0.01, 6.91]
Boyer et al, 2009	23	32	1	1	0.0%	0.82 [0.03, 22.09]
Chao et al, 2013	20	90	15	31	15.3%	0.30 [0.13, 0.72]
Corona et al, 2016	6	18	0	2	0.0%	2.60 [0.11, 62.57]
George et al, 2009	8	61	0	11	2.5%	3.65 [0.20, 67.92]
Huang et al, 2008	2	14	1	2	0.0%	0.17 [0.01, 3.89]
Huang et al, 2011	38	280	19	192	20.8%	1.43 [0.80, 2.56]
Kaiser et al, 1981	6	17	2	3	3.1%	0.27 [0.02, 3.67]
Kalaivani et al, 2012	10	35	5	25	10.2%	1.60 [0.47, 5.44]
Lee et al, 2014	12	81	6	19	11.1%	0.38 [0.12, 1.18]
Nawijn et al, Feb 2019	14	51	7	20	11.6%	0.70 [0.23, 2.12]
Ogilvie et al, 2006	13	134	1	16	4.5%	1.61 [0.20, 13.21]
Palmer et al, 1995	10	21	3	9	0.0%	1.82 [0.36, 9.27]
Park et al, 2016	4	20	3	10	6.1%	0.58 [0.10, 3.33]
Stephenson et al, 1992	2	7	12	22	0.0%	0.33 [0.05, 2.10]
Wang et al, 1992	3	14	3	4	0.0%	0.09 [0.01, 1.22]
Yu et al, 2004	2	10	0	3	2.0%	2.06 [0.08, 54.80]
**Total (95% CI)**		**817**		**398**	**100.0%**	**0.85 [0.53, 1.38]**
Total events	135		68			

Heterogeneity: Tau² = 0.20; Chi² = 15.31, df = 10 (P = 0.12); I² = 35%
Test for overall effect: Z = 0.64 (P = 0.52)

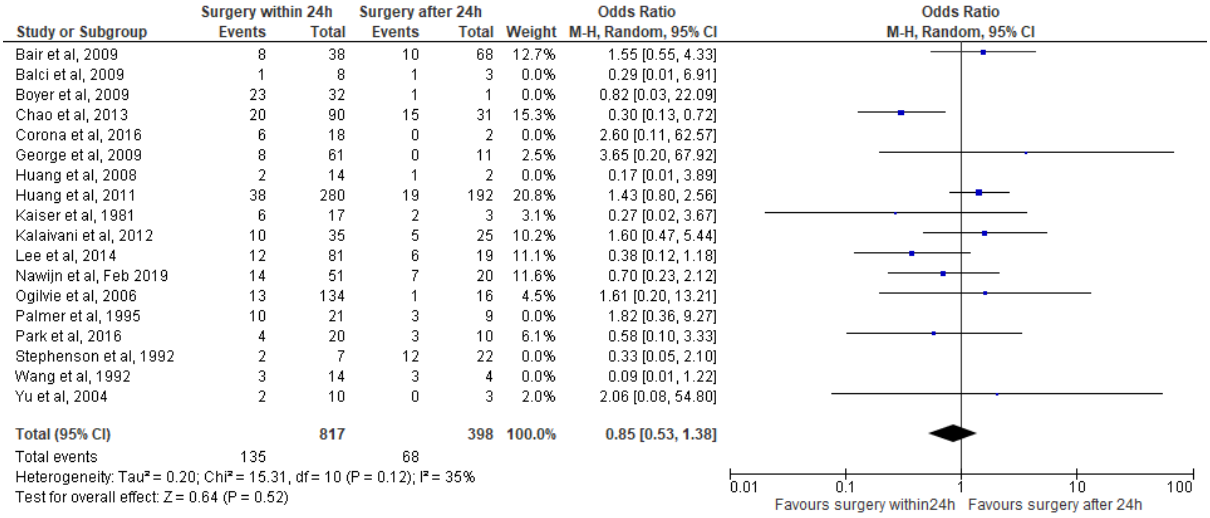

Subgroup analysis: Excluding studies limited to specific microbial types of NSTI

Study or Subgroup	Surgery within 24h Events	Surgery within 24h Total	Surgery after 24h Events	Surgery after 24h Total	Weight	Odds Ratio M-H, Random, 95% CI
Bair et al, 2009	8	38	10	68	12.6%	1.55 [0.55, 4.33]
Balci et al, 2009	1	8	1	3	1.3%	0.29 [0.01, 6.91]
Boyer et al, 2009	23	32	1	1	1.2%	0.82 [0.03, 22.09]
Chao et al, 2013	20	90	15	31	0.0%	0.30 [0.13, 0.72]
Corona et al, 2016	6	18	0	2	1.3%	2.60 [0.11, 62.57]
George et al, 2009	8	61	0	11	1.6%	3.65 [0.20, 67.92]
Huang et al, 2008	2	14	1	2	1.3%	0.17 [0.01, 3.89]
Huang et al, 2011	38	280	19	192	39.1%	1.43 [0.80, 2.56]
Kaiser et al, 1981	6	17	2	3	2.0%	0.27 [0.02, 3.67]
Kalaivani et al, 2012	10	35	5	25	8.9%	1.60 [0.47, 5.44]
Lee et al, 2014	12	81	6	19	0.0%	0.38 [0.12, 1.18]
Nawijn et al, Feb 2019	14	51	7	20	10.9%	0.70 [0.23, 2.12]
Ogilvie et al, 2006	13	134	1	16	3.0%	1.61 [0.20, 13.21]
Palmer et al, 1995	10	21	3	9	5.0%	1.82 [0.36, 9.27]
Park et al, 2016	4	20	3	10	4.4%	0.58 [0.10, 3.33]
Stephenson et al, 1992	2	7	12	22	3.9%	0.33 [0.05, 2.10]
Wang et al, 1992	3	14	3	4	2.0%	0.09 [0.01, 1.22]
Yu et al, 2004	2	10	0	3	1.2%	2.06 [0.08, 54.80]
**Total (95% CI)**		**760**		**391**	**100.0%**	**1.11 [0.77, 1.60]**
Total events	150		68			

Heterogeneity: Tau² = 0.00; Chi² = 12.61, df = 15 (P = 0.63); I² = 0%
Test for overall effect: Z = 0.54 (P = 0.59)

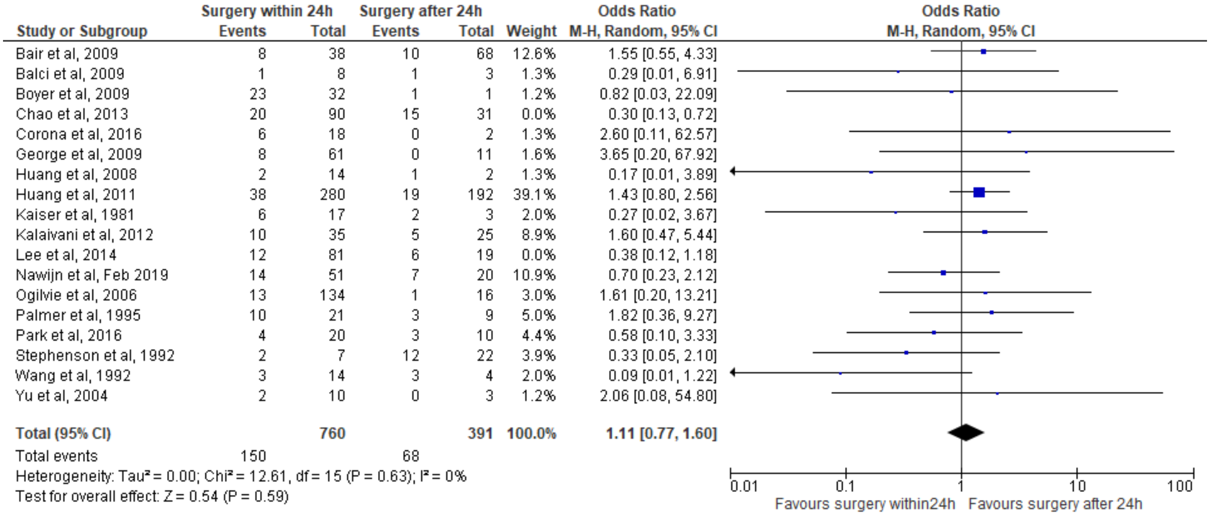

Surgery within 24 hours after presentation - amputation as outcome

Study or Subgroup	Surgery within 24h Events	Surgery within 24h Total	Surgery after 24h Events	Surgery after 24h Total	Weight	Odds Ratio M-H, Random, 95% CI
Corona et al, 2016	4	18	0	2	12.6%	1.55 [0.06, 38.65]
Huang et al, 2008	4	14	0	2	12.5%	2.14 [0.08, 54.22]
Kaiser et al, 1981	0	8	1	2	10.0%	0.06 [0.00, 2.24]
Mittapalli et al, 2015	3	12	2	12	30.1%	1.67 [0.22, 12.35]
Nawijn et al, Feb 2019	4	25	3	7	34.9%	0.25 [0.04, 1.60]
**Total (95% CI)**		**77**		**25**	**100.0%**	**0.63 [0.20, 2.05]**
Total events	15		6			

Heterogeneity: Tau² = 0.15; Chi² = 4.34, df = 4 (P = 0.36); I² = 8%
Test for overall effect: Z = 0.76 (P = 0.45)

Odds Ratio
M-H, Random, 95% CI

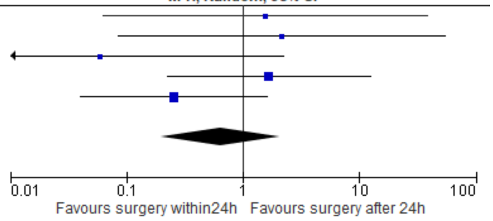

Subgroup analysis: High-quality studies

Study or Subgroup	Surgery within 24h Events	Surgery within 24h Total	Surgery after 24h Events	Surgery after 24h Total	Weight	Odds Ratio M-H, Random, 95% CI
Corona et al, 2016	4	18	0	2	0.0%	1.55 [0.06, 38.65]
Huang et al, 2008	4	14	0	2	0.0%	2.14 [0.08, 54.22]
Kaiser et al, 1981	0	8	1	2	0.0%	0.06 [0.00, 2.24]
Mittapalli et al, 2015	3	12	2	12	0.0%	1.67 [0.22, 12.35]
Nawijn et al, Feb 2019	4	25	3	7	100.0%	0.25 [0.04, 1.60]
**Total (95% CI)**		**25**		**7**	**100.0%**	**0.25 [0.04, 1.60]**
Total events	4		3			

Heterogeneity: Not applicable
Test for overall effect: Z = 1.46 (P = 0.14)

Odds Ratio
M-H, Random, 95% CI

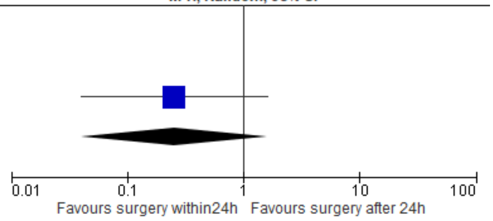

Subgroup analysis: Studies published ≥2009

Study or Subgroup	Surgery within 24h Events	Surgery within 24h Total	Surgery after 24h Events	Surgery after 24h Total	Weight	Odds Ratio M-H, Random, 95% CI
Corona et al, 2016	4	18	0	2	15.8%	1.55 [0.06, 38.65]
Huang et al, 2008	4	14	0	2	0.0%	2.14 [0.08, 54.22]
Kaiser et al, 1981	0	8	1	2	0.0%	0.06 [0.00, 2.24]
Mittapalli et al, 2015	3	12	2	12	38.8%	1.67 [0.22, 12.35]
Nawijn et al, Feb 2019	4	25	3	7	45.4%	0.25 [0.04, 1.60]
**Total (95% CI)**		**55**		**21**	**100.0%**	**0.70 [0.19, 2.58]**
Total events	11		5			

Heterogeneity: Tau² = 0.09; Chi² = 2.13, df = 2 (P = 0.34); I² = 6%
Test for overall effect: Z = 0.53 (P = 0.59)

Odds Ratio
M-H, Random, 95% CI

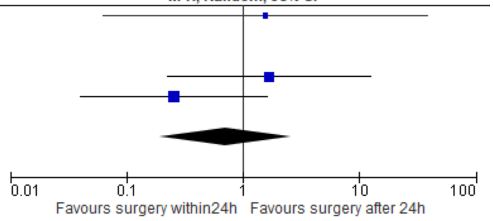

Subgroup analysis: Studies without limitation on affected body region by NSTI

Study or Subgroup	Surgery within 24h Events	Surgery within 24h Total	Surgery after 24h Events	Surgery after 24h Total	Weight	Odds Ratio M-H, Random, 95% CI
Corona et al, 2016	4	18	0	2	0.0%	1.55 [0.06, 38.65]
Huang et al, 2008	4	14	0	2	0.0%	2.14 [0.08, 54.22]
Kaiser et al, 1981	0	8	1	2	17.4%	0.06 [0.00, 2.24]
Mittapalli et al, 2015	3	12	2	12	39.4%	1.67 [0.22, 12.35]
Nawijn et al, Feb 2019	4	25	3	7	43.1%	0.25 [0.04, 1.60]
**Total (95% CI)**		**45**		**21**	**100.0%**	**0.41 [0.08, 2.26]**
Total events	7		6			

Heterogeneity: Tau² = 0.86; Chi² = 3.22, df = 2 (P = 0.20); I² = 38%
Test for overall effect: Z = 1.02 (P = 0.31)

Odds Ratio
M-H, Random, 95% CI

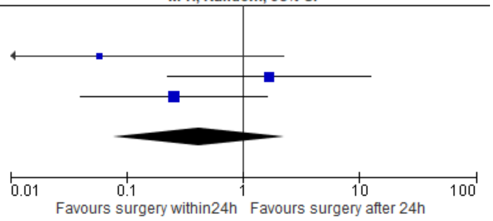

Subgroup analysis: Excluding studies limited to specific microbial types of NSTI

Study or Subgroup	Surgery within 24h Events	Surgery within 24h Total	Surgery after 24h Events	Surgery after 24h Total	Weight	Odds Ratio M-H, Random, 95% CI
Corona et al, 2016	4	18	0	2	15.6%	1.55 [0.06, 38.65]
Huang et al, 2008	4	14	0	2	0.0%	2.14 [0.08, 54.22]
Kaiser et al, 1981	0	8	1	2	12.5%	0.06 [0.00, 2.24]
Mittapalli et al, 2015	3	12	2	12	33.7%	1.67 [0.22, 12.35]
Nawijn et al, Feb 2019	4	25	3	7	38.1%	0.25 [0.04, 1.60]
**Total (95% CI)**		**63**		**23**	**100.0%**	**0.53 [0.14, 2.06]**
Total events	11		6			

Heterogeneity: Tau² = 0.38; Chi² = 3.71, df = 3 (P = 0.29); I² = 19%
Test for overall effect: Z = 0.92 (P = 0.36)

Odds Ratio
M-H, Random, 95% CI

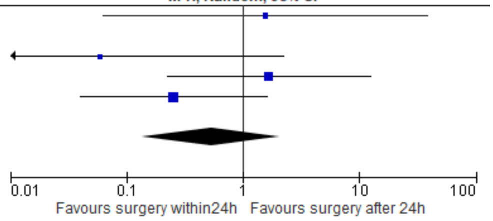

Surgery within 3 days after onset symptoms - mortality as outcome

Study or Subgroup	Surgery within 3 days Events	Surgery within 3 days Total	Surgery after 3 days Events	Surgery after 3 days Total	Weight	Odds Ratio M-H, Random, 95% CI
Knutson et al, 1983	4	6	2	3	11.5%	1.00 [0.05, 18.91]
Lee et al, 2014	11	78	6	21	76.6%	0.41 [0.13, 1.29]
Liu et al, 2012	0	14	10	50	11.9%	0.13 [0.01, 2.42]
**Total (95% CI)**		**98**		**74**	**100.0%**	**0.40 [0.15, 1.08]**
Total events	15		18			

Heterogeneity: Tau² = 0.00; Chi² = 0.98, df = 2 (P = 0.61); I² = 0%
Test for overall effect: Z = 1.81 (P = 0.07)

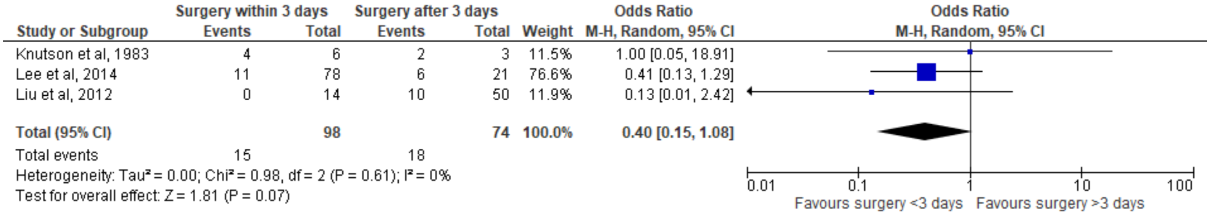

Subgroup analysis: High-quality studies

Study or Subgroup	Surgery within 3 days Events	Surgery within 3 days Total	Surgery after 3 days Events	Surgery after 3 days Total	Weight	Odds Ratio M-H, Random, 95% CI
Knutson et al, 1983	4	6	2	3		Not estimable
Lee et al, 2014	11	78	6	21	100.0%	0.41 [0.13, 1.29]
Liu et al, 2012	0	14	10	50	0.0%	0.13 [0.01, 2.42]
**Total (95% CI)**		**78**		**21**	**100.0%**	**0.41 [0.13, 1.29]**
Total events	11		6			

Heterogeneity: Not applicable
Test for overall effect: Z = 1.53 (P = 0.13)

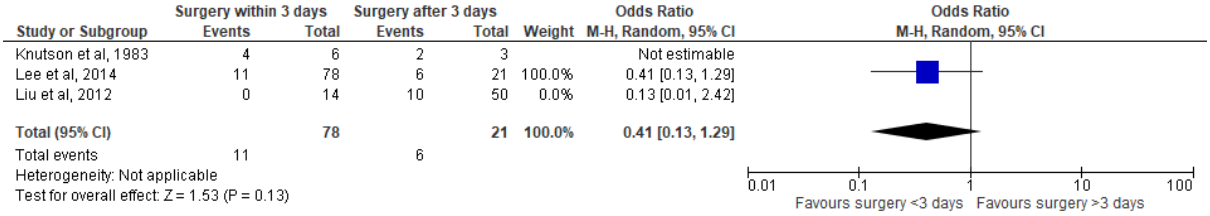

Subgroup analysis: Studies published ≥2009

Study or Subgroup	Surgery within 3 days Events	Surgery within 3 days Total	Surgery after 3 days Events	Surgery after 3 days Total	Weight	Odds Ratio M-H, Random, 95% CI
Knutson et al, 1983	4	6	2	3	0.0%	1.00 [0.05, 18.91]
Lee et al, 2014	11	78	6	21	86.6%	0.41 [0.13, 1.29]
Liu et al, 2012	0	14	10	50	13.4%	0.13 [0.01, 2.42]
**Total (95% CI)**		**92**		**71**	**100.0%**	**0.35 [0.12, 1.02]**
Total events	11		16			

Heterogeneity: Tau² = 0.00; Chi² = 0.56, df = 1 (P = 0.45); I² = 0%
Test for overall effect: Z = 1.92 (P = 0.05)

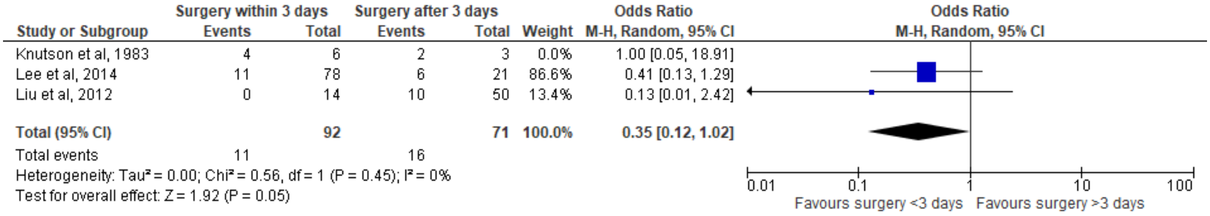

Subgroup analysis: Studies without limitation on affected body region by NSTI

Study or Subgroup	Surgery within 3 days Events	Surgery within 3 days Total	Surgery after 3 days Events	Surgery after 3 days Total	Weight	Odds Ratio M-H, Random, 95% CI
Knutson et al, 1983	4	6	2	3	13.1%	1.00 [0.05, 18.91]
Lee et al, 2014	11	78	6	21	86.9%	0.41 [0.13, 1.29]
Liu et al, 2012	0	14	10	50	0.0%	0.13 [0.01, 2.42]
**Total (95% CI)**		**84**		**24**	**100.0%**	**0.46 [0.16, 1.34]**
Total events	15		8			

Heterogeneity: Tau² = 0.00; Chi² = 0.31, df = 1 (P = 0.58); I² = 0%
Test for overall effect: Z = 1.43 (P = 0.15)

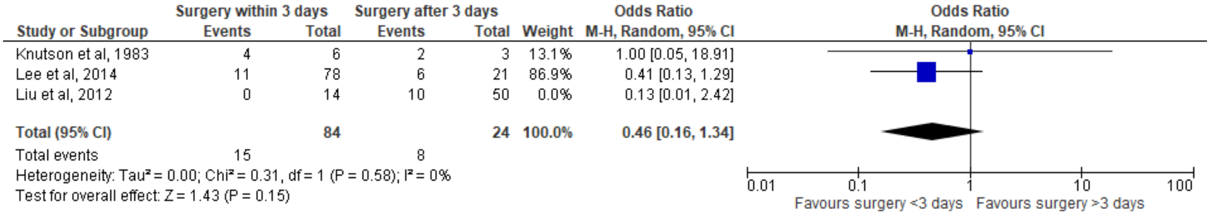

Subgroup analysis: Excluding studies limited to specific microbial types of NSTI

Study or Subgroup	Surgery within 3 days Events	Surgery within 3 days Total	Surgery after 3 days Events	Surgery after 3 days Total	Weight	Odds Ratio M-H, Random, 95% CI
Knutson et al, 1983	4	6	2	3	0.0%	1.00 [0.05, 18.91]
Lee et al, 2014	11	78	6	21	0.0%	0.41 [0.13, 1.29]
Liu et al, 2012	0	14	10	50	100.0%	0.13 [0.01, 2.42]
**Total (95% CI)**		**14**		**50**	**100.0%**	**0.13 [0.01, 2.42]**
Total events	0		10			

Heterogeneity: Not applicable
Test for overall effect: Z = 1.36 (P = 0.17)

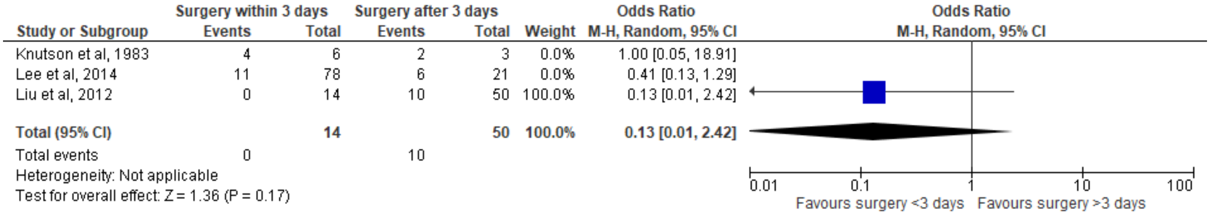

Hospital presentation within 3 days after onset symptoms - mortality as outcome

Study or Subgroup	Presentation within 3days Events	Presentation within 3days Total	Presentation after 3 days Events	Presentation after 3 days Total	Weight	Odds Ratio M-H, Random, 95% CI
Barupal et al, 2019	7	15	9	35	19.0%	2.53 [0.71, 8.97]
Boyer et al, 2009	25	65	18	41	22.6%	0.80 [0.36, 1.77]
Catena et al, 2004	1	4	6	7	8.3%	0.06 [0.00, 1.23]
Ferretti et al, 2017	3	14	0	6	8.2%	3.96 [0.18, 89.19]
Huang et al, 2008	1	14	2	2	7.1%	0.02 [0.00, 0.72]
Knutson et al, 1983	5	8	1	1	7.1%	0.52 [0.02, 16.83]
Lee et al, 2014	12	90	5	9	17.6%	0.12 [0.03, 0.52]
Yu et al, 2004	1	6	2	9	10.1%	0.70 [0.05, 10.01]
**Total (95% CI)**		**216**		**110**	**100.0%**	**0.49 [0.16, 1.44]**
Total events	55		43			

Heterogeneity: Tau² = 1.19; Chi² = 17.11, df = 7 (P = 0.02); I² = 59%
Test for overall effect: Z = 1.30 (P = 0.19)

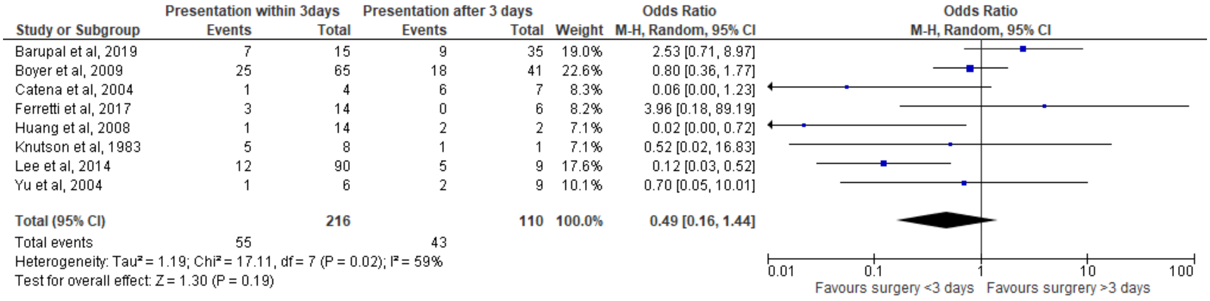

Subgroup analysis: High-quality studies

Study or Subgroup	Presentation within 3days Events	Presentation within 3days Total	Presentation after 3 days Events	Presentation after 3 days Total	Weight	Odds Ratio M-H, Random, 95% CI
Barupal et al, 2019	7	15	9	35	32.2%	2.53 [0.71, 8.97]
Boyer et al, 2009	25	65	18	41	37.8%	0.80 [0.36, 1.77]
Catena et al, 2004	1	4	6	7	0.0%	0.06 [0.00, 1.23]
Ferretti et al, 2017	3	14	0	6	0.0%	3.96 [0.18, 89.19]
Huang et al, 2008	1	14	2	2	0.0%	0.02 [0.00, 0.72]
Knutson et al, 1983	5	8	1	1		Not estimable
Lee et al, 2014	12	90	5	9	30.0%	0.12 [0.03, 0.52]
Yu et al, 2004	1	6	2	9	0.0%	0.70 [0.05, 10.01]
**Total (95% CI)**		**170**		**85**	**100.0%**	**0.66 [0.15, 2.83]**
Total events	44		32			

Heterogeneity: Tau² = 1.29; Chi² = 9.56, df = 2 (P = 0.008); I² = 79%
Test for overall effect: Z = 0.56 (P = 0.58)

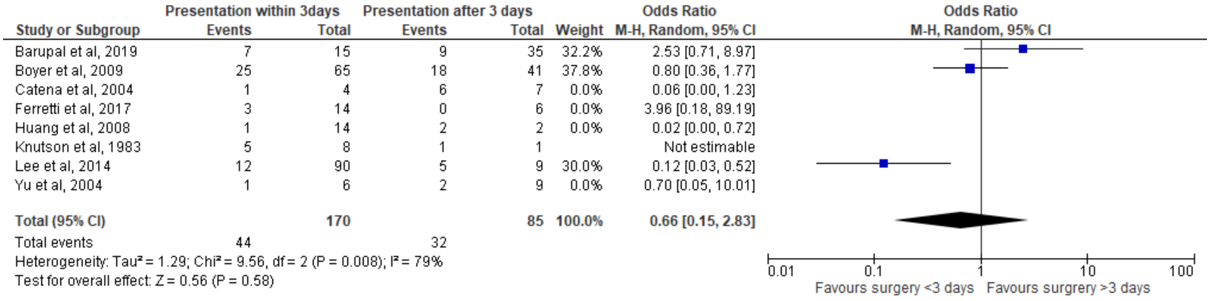

Subgroup analysis: Studies published ≥2009

Study or Subgroup	Presentation within 3days Events	Presentation within 3days Total	Presentation after 3 days Events	Presentation after 3 days Total	Weight	Odds Ratio M-H, Random, 95% CI
Barupal et al, 2019	7	15	9	35	28.2%	2.53 [0.71, 8.97]
Boyer et al, 2009	25	65	18	41	33.5%	0.80 [0.36, 1.77]
Catena et al, 2004	1	4	6	7	0.0%	0.06 [0.00, 1.23]
Ferretti et al, 2017	3	14	0	6	12.2%	3.96 [0.18, 89.19]
Huang et al, 2008	1	14	2	2	0.0%	0.02 [0.00, 0.72]
Knutson et al, 1983	5	8	1	1	0.0%	0.52 [0.02, 16.83]
Lee et al, 2014	12	90	5	9	26.1%	0.12 [0.03, 0.52]
Yu et al, 2004	1	6	2	9	0.0%	0.70 [0.05, 10.01]
**Total (95% CI)**		**184**		**91**	**100.0%**	**0.82 [0.22, 3.09]**
Total events	47		32			

Heterogeneity: Tau² = 1.20; Chi² = 10.64, df = 3 (P = 0.01); I² = 72%
Test for overall effect: Z = 0.29 (P = 0.78)

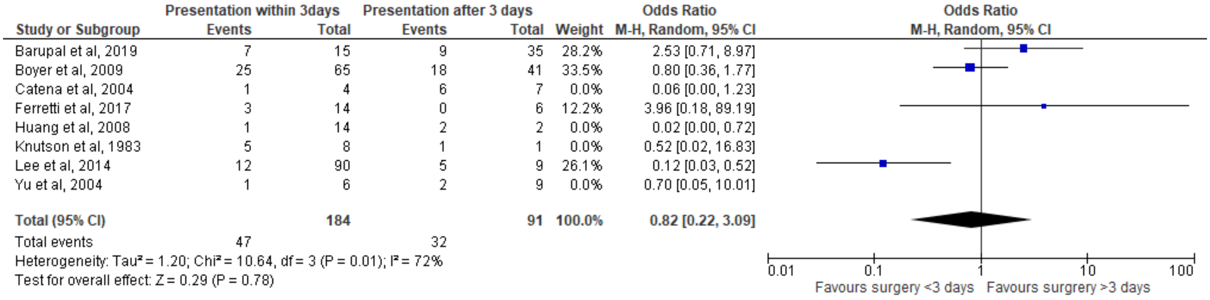

Subgroup analysis: Studies without limitation on affected body region by NSTI

Study or Subgroup	Presentation within 3days Events	Presentation within 3days Total	Presentation after 3 days Events	Presentation after 3 days Total	Weight	Odds Ratio M-H, Random, 95% CI
Barupal et al, 2019	7	15	9	35	27.6%	2.53 [0.71, 8.97]
Boyer et al, 2009	25	65	18	41	0.0%	0.80 [0.36, 1.77]
Catena et al, 2004	1	4	6	7	15.1%	0.06 [0.00, 1.23]
Ferretti et al, 2017	3	14	0	6	0.0%	3.96 [0.18, 89.19]
Huang et al, 2008	1	14	2	2	0.0%	0.02 [0.00, 0.72]
Knutson et al, 1983	5	8	1	1	13.3%	0.52 [0.02, 16.83]
Lee et al, 2014	12	90	5	9	26.3%	0.12 [0.03, 0.52]
Yu et al, 2004	1	6	2	9	17.6%	0.70 [0.05, 10.01]
**Total (95% CI)**		**123**		**61**	**100.0%**	**0.41 [0.08, 2.13]**
Total events	26		23			

Heterogeneity: Tau² = 2.10; Chi² = 11.84, df = 4 (P = 0.02); I² = 66%
Test for overall effect: Z = 1.06 (P = 0.29)

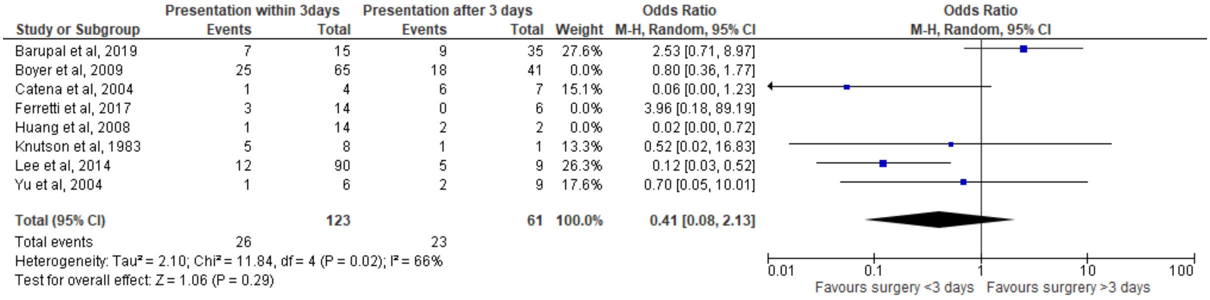

Subgroup analysis: Excluding studies limited to specific microbial types of NSTI

Study or Subgroup	Presentation within 3days Events	Total	Presentation after 3 days Events	Total	Weight	Odds Ratio M-H, Random, 95% CI
Barupal et al, 2019	7	15	9	35	29.6%	2.53 [0.71, 8.97]
Boyer et al, 2009	25	65	18	41	41.5%	0.80 [0.36, 1.77]
Catena et al, 2004	1	4	6	7	8.8%	0.06 [0.00, 1.23]
Ferretti et al, 2017	3	14	0	6	8.8%	3.96 [0.18, 89.19]
Huang et al, 2008	1	14	2	2	0.0%	0.02 [0.00, 0.72]
Knutson et al, 1983	5	8	1	1	0.0%	0.52 [0.02, 16.83]
Lee et al, 2014	12	90	5	9	0.0%	0.12 [0.03, 0.52]
Yu et al, 2004	1	6	2	9	11.3%	0.70 [0.05, 10.01]
**Total (95% CI)**		**104**		**98**	**100.0%**	**1.01 [0.37, 2.74]**
Total events	37		35			

Heterogeneity: Tau² = 0.47; Chi² = 6.53, df = 4 (P = 0.16); I² = 39%
Test for overall effect: Z = 0.01 (P = 0.99)

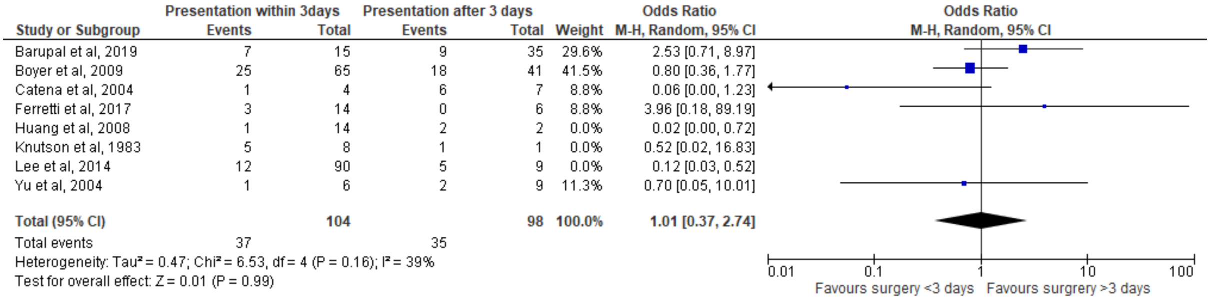

Supplement: Supplementary file 4 — Additional file 4: Forest plots for all (subgroup) analyses assessing surgical timing in relation to mortality and amputation due to necrotizing soft tissue infections. [file 13017_2019_286_MOESM4_ESM.pdf]
