# Supplementary material for: Time is of the essence when treating necrotizing soft tissue infections: a systematic review and meta-analysis
Source: World J Emerg Surg. 2020 Jan 8;15:4. doi: 10.1186/s13017-019-0286-6 (PMC6950871; doi:10.1186/s13017-019-0286-6)
Supplement: Supplementary file 5 — Additional file 5: Results from quality assessment of articles included in meta-analyses assessing surgical timing in relation to mortality and amputation due to necrotizing soft tissue infections. [file 13017_2019_286_MOESM5_ESM.pdf]

Additional file 5 Results from quality assessment of articles included in meta-analyses assessing surgical timing in relation to mortality and amputation due to necrotizing soft tissue infections

Study	Prospective collection of data	Inclusion and exclusion criteria	Diagnostic criteria used	Definition of the outcome	Total
Bair et al. 2009	1	1	2	1	5
Balci et al. 2018	1	1	2	1	5
Barupal et al. 2019	2	2	1	2	7
Boyer et al. 2009	1	2	2	2	7
Catena et al. 2004	0	1	0	0	1
Chao et al. 2013	1	2	2	2	7
Corona et al. 2016	1	1	2	1	5
Ferretti et al. 2017	1	1	0	0	2
George et al. 2009	1	1	0	2	4
Hadeed et al. 2016	1	1	2	2	6
Huang et al. 2008	0	1	2	1	4
Huang et al. 2011	1	1	2	2	6
Kaiser et al. 1981	1	1	0	0	2
Kalaivani et al. 2012	1	1	1	1	4
Knutson et al. 1983	0	2	2	0	4
Kobayashi et al. 2011	1	1	2	2	6
Latifi et al. 2018	1	2	2	2	7
Lee et al. 2014	1	1	2	2	6
Lille et al. 1996	1	2	2	0	5
Liu et al. 2012	1	1	0	1	3
Mittapalli et al. 2015	1	2	0	2	5
Nawijn et al. Aug 2019	2	2	2	2	8
Nawijn et al. Feb 2019	1	2	2	2	7
Ogilvie et al. 2006	1	1	2	1	5
Pakula et al. 2012	1	1	2	1	5
Palmer et al. 1995	0	1	0	1	2
Park et al. 2016	1	1	0	2	4
Stephenson et al. 1992	1	2	0	0	3
Sudarsky et al. 1987	1	1	2	0	4
Tsai et al. 2010	1	1	0	2	4
Tsai et al. 2015	1	2	2	0	5
Wang et al. 1992	1	1	2	0	4
Yu et al. 2004	1	1	0	0	2
